# Oxidation-regulated wide-temperature tribological behavior and mechanism of a Mo/Mo tribopair lubricated by Ga-based liquid metal

**DOI:** 10.1016/j.isci.2026.117805

**Published:** 2026-10-09

**Authors:** Qiaona Wang, Hongji Liu, Guohua Zhang, Tongyang Li, Hailong Cui, Xing Feng, Yuan Yu, Zhengfeng Ma, Xiaoling Wang, Zhuhui Qiao, Huaguo Tang

**Affiliations:** 1 State Key Laboratory of Solid Lubrication, Lanzhou Institute of Chemical Physics, Chinese Academy of Sciences, Lanzhou 730000, P.R. China; 2 Center of Materials Science and Optoelectronics Engineering, University of Chinese Academy of Sciences, Beijing 100049, P.R. China; 3 College of Materials Science and Engineering, Hebei University of Engineering, Handan 056038, P.R. China; 4 Sichuan Precision and Ultra-Precision Machining Engineering Technology Center, Sichuan 610200, P.R. China; 5 Shandong Laboratory of Advanced Materials and Green Manufacturing at Yantai, Yantai 264006, P.R. China; 6 School of Mechanical and Aerospace Engineering, Nanyang Technological University, Singapore 639798, Singapore

**Keywords:** gallium-based liquid metal, Mo/Mo tribopair, wide-temperature lubrication, tribo-oxidation, Ga-O-rich tribofilm, Ga-associated interfacial weakening

## Abstract

Gallium-based liquid metals combine fluidity with high thermal and electrical conductivity, making them attractive for lubrication in demanding environments. We investigated the tribological behavior of a Mo/Mo self-mated pair lubricated with Ga-In-Sn (GIS) from room temperature to 300°C in air and argon. Friction and wear tests, together with energy-dispersive spectroscopy (EDS) and X-ray photoelectron spectroscopy (XPS) analyses, were used to examine interfacial behavior and tribofilm formation. In air, GIS reduced the Mo-disk wear rate by 90.9%–99.4% compared with the unlubricated pair, accompanied by the formation of a Ga-O-rich tribofilm. In argon, localized Ga retention, tribofilm delamination, and lamellar spalling became more pronounced at elevated temperature, leading to a wear rate of 15.6 × 10^−5^ mm^3^/(N·m) at 300°C. These results provide insights into the effects of environmental conditions on liquid-metal lubrication and support the selection of suitable lubrication conditions for high-temperature sliding systems.

## Highlights

• GIS lubricates Mo/Mo tribopairs from room temperature to 300°C
• In air, GIS reduces the Mo-disk wear rate by 90.9%–99.4%
• A Ga-O-rich tribofilm enables liquid-metal/oxide composite lubrication
• Oxide-film protection competes with Ga-associated interfacial weakening

## Graphical abstract

**Figure:**
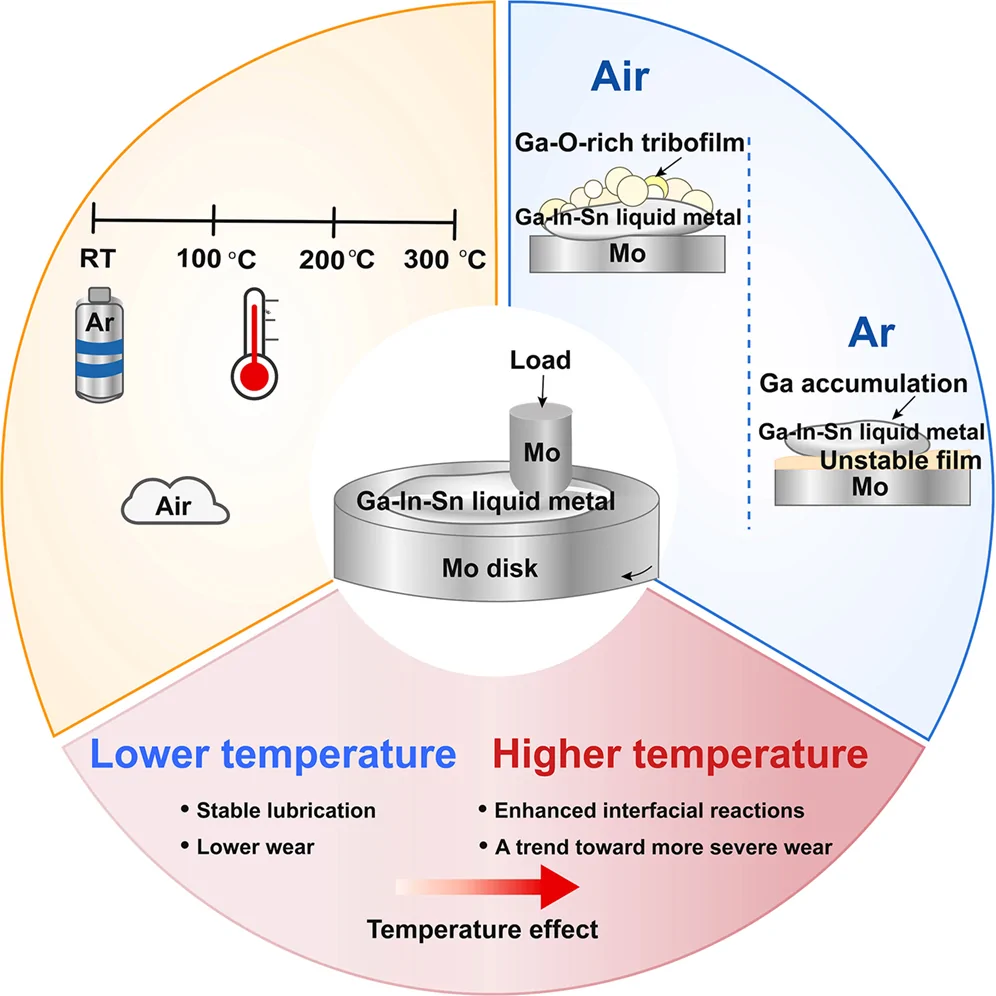

## Teaser

Interface science; Engineering materials; Tribology

## Introduction

With the rapid development of advanced medical equipment, nuclear-industry devices, and high-power X-ray sources toward higher precision, higher power density, and longer service life, the lubrication stability of key sliding bearings has become a critical factor governing reliable system operation.^1^ Such bearings usually operate under complex conditions involving elevated temperature, high contact stress, strong irradiation, and confined spaces. The lubricant is therefore required not only to maintain low friction and low wear but also to provide excellent thermal conductivity, electrical conductivity, and low volatility.^2^ Conventional lubricating oils and greases are prone to evaporation, thermal decomposition, and oxidative aging under high-temperature or vacuum conditions, which lead to viscosity instability, carbonaceous deposits, and lubricant-film failure.^3^ Meanwhile, their intrinsic thermal and electrical conductivities are limited, making it difficult to satisfy the coupled requirements of thermal management and functional lubrication in precision bearings for high-energy radiation equipment.^4^ Traditional liquid metals such as lead and mercury possess high thermal and electrical conductivity, but their toxicity and environmental risks severely restrict their engineering use.^5^ In contrast, gallium-based liquid metals (GLMs) combine metallic thermal/electrical transport with liquid-state fluidity and show the advantages of low melting point, low saturated vapor pressure, wide-temperature stability, and relatively low toxicity, offering functional lubrication options for demanding service conditions.^6^^,^^7^^,^^8^^,^^9^

Recent studies on GLM lubrication have shown that its friction-reducing and anti-wear performance mainly originates from liquid-metal hydrodynamic or fluid lubrication, rapid interfacial spreading, and reaction/transfer films formed *in situ* during sliding.^10^ For Cu/Al tribopairs, Ma et al. reported that Ga-In-Sn (GIS) lubrication reduced the friction coefficient by approximately 1.5–3 times and the wear rate of the Cu alloy by approximately 4–16 times compared with dry sliding, with more pronounced lubrication under high-load and high-speed conditions.^11^ Zhang et al. further demonstrated, using current-carrying Cu/Al tribopairs, that GLM stabilized the friction coefficient at 0.19–0.27 and reduced the wear rate of the Cu alloy by approximately 3.1–8.3 times; its high electrical conductivity also helped suppress arc ablation.^12^ Studies under high-temperature vacuum conditions for space applications have also shown that GLM can form a continuous Ga-Al metallic tribofilm *in situ* on Cu alloy surfaces. This film acts synergistically with the intrinsic fluid lubrication of the liquid metal, resulting in significantly lower friction and wear than dry sliding.^13^ For steel/ceramic and steel/steel tribopairs, GLM has been reported to form Ga-rich films, FeGa_3_ intermetallic compounds, or Ga-oxide-containing boundary lubrication films on worn surfaces, thereby separating real contact areas and reducing adhesive and abrasive wear. The study by Guo et al. on the T91 steel/GCr15 friction pair shows that the introduction of GLM can effectively reduce the friction coefficient and wear rate.^14^ After applying an electric current, the electric field effect promotes the adsorption of Ga elements on the cathode surface and inhibits their oxidation, further lowering the wear loss to 56% of that under the current-free condition.^15^ These findings collectively indicate that GLM lubrication does not simply rely on a low-shear liquid layer but is strongly governed by the tribopair material, interfacial reactions, and tribofilm stability.

To further improve the load-carrying capacity and interfacial adaptability of GLMs, several strategies have been proposed, including metallic-element regulation and the incorporation of inorganic nano-lubricating phases. Yu et al. modified GIS by Cu doping, generating CuGa_2_ phases *in situ* in the liquid metal and improving wettability, heat transfer, and tribofilm structure; in particular, GC0.2 reduced the wear rate of T91 steel at 150°C by 72.95% compared with pure GLM lubrication.^16^ Metallic additives such as Ag, Al, and Bi have also been used to regulate the oxidation behavior, reaction-film structure, and high-temperature lubrication stability of GLMs.^17^^,^^18^^,^^19^ In parallel, inorganic nanoparticles such as h-BN, MoSe_2_, and WSe_2_ can increase the viscosity and extreme-pressure load-carrying capacity of liquid metals and construct composite tribofilms containing Ga oxides, metal oxides, and layered lubricating phases at the friction interface.^20^^,^^21^ Notably, MoSe_2_/WSe_2_-reinforced GIS increases the extreme-pressure load-carrying capacity of liquid metal from approximately 1,600 to over 2,000 N, indicating that the synergistic design of a “liquid-metal matrix-nano-reinforcement-tribochemical film” can contribute to improved GLM lubrication performance.^22^ In addition, surface texturing can store liquid metal and promote Ga-film deposition, providing an effective strategy for improving lubricant supply stability and wear resistance.^23^

Although these studies have demonstrated the lubrication potential of GLMs for Cu alloys, Al alloys, T91 steel, GCr15 steel, Inconel alloys, and other materials, their interfacial mechanisms are highly material-selective. For precision sliding bearings used in high-energy radiation devices and high-power X-ray sources, material selection is not determined solely by mechanical properties and lubricant compatibility; it is also strictly constrained by the thermal expansion coefficient, dimensional stability, and high-temperature strength. Mo and Mo-based alloys have high melting points, high hardness, excellent high-temperature strength, and low thermal expansion coefficients, enabling good dimensional matching from room temperature (RT) to 300°C.^24^ They are therefore important candidate materials for this class of precision sliding bearings. However, the wetting, adsorption, oxidation, and interfacial reaction behaviors between a Mo/Mo self-mated tribopair and GLM differ from those reported for Cu/Al or steel/ceramic tribopairs. Recent studies have indicated that the interfacial interaction between Mo and Ga-based liquid metals is temperature dependent, with no obvious Mo-Ga reaction detected within the relatively low-temperature range.^25^^,^^26^ However, under sliding conditions, Ga may accumulate in mechanically damaged near-surface regions and influence the local interfacial state, while the oxidative atmosphere can further affect the formation and stability of Ga-O-containing tribofilms.^27^ Therefore, clarifying the wide-temperature tribological behavior and atmosphere-regulated mechanism of Mo/Mo tribopairs lubricated by GLM is essential for expanding the application of GLMs in precision bearings.

Based on this background, this work focuses on a Mo/Mo self-mated tribopair for sliding-bearing applications in high-energy radiation devices and high-power X-ray sources. The lubrication performance of GIS liquid metal is systematically evaluated from RT to 300°C, and the friction and wear responses in air and argon atmospheres are compared. This study provides three main contributions. First, GLM lubrication is extended to a low-thermal-expansion and high-temperature-stable Mo/Mo self-mated tribopair that is relevant to practical precision-bearing material systems. Second, the effects of atmospheric oxidation on GLM-lubricated Mo surfaces are examined: Ga oxidation in air promotes a liquid-metal/Ga-O-rich composite tribofilm, whereas restricted oxidation in argon favors localized Ga retention and an unstable, discontinuous interfacial layer. Third, competition between oxide-film protection and Ga-associated near-surface weakening is proposed for the Mo/Mo-GIS interface over a wide temperature range, while direct observations are distinguished from mechanistic interpretation.

## Results

### Tribological performance

Before the tribological tests, the as-prepared GIS was characterized by scanning electron microscopy (SEM)/EDS and X-ray diffraction (XRD). The SEM image and elemental maps show a homogeneous micrometer-scale distribution of Ga, In, and Sn without obvious elemental segregation (Figure S1), while the XRD pattern exhibits a broad diffuse scattering feature centered at approximately 35° without resolvable sharp Bragg peaks (Figure S2), indicating no detectable long-range crystalline order.^28^

Figure 1 shows the friction coefficient curves of the Mo/Mo tribopair under dry sliding in air, GIS lubrication in air, and GIS lubrication in argon at different temperatures. Figure 2 summarizes the corresponding average friction coefficients. Under dry sliding in air, the Mo/Mo self-mated tribopair exhibits high and strongly fluctuating friction coefficients, indicating severe adhesion, plowing, and local spalling at the friction interface. The average friction coefficient is approximately 0.57 at RT, increases to approximately 0.65 at 100°C and 300°C, and decreases to approximately 0.51 at 200°C. Therefore, increasing temperature alone does not substantially reduce the interfacial shear resistance under dry sliding; instead, temperature influences friction stability by changing oxide-layer formation and spalling behavior.^29^^,^^30^

**Figure 1:**
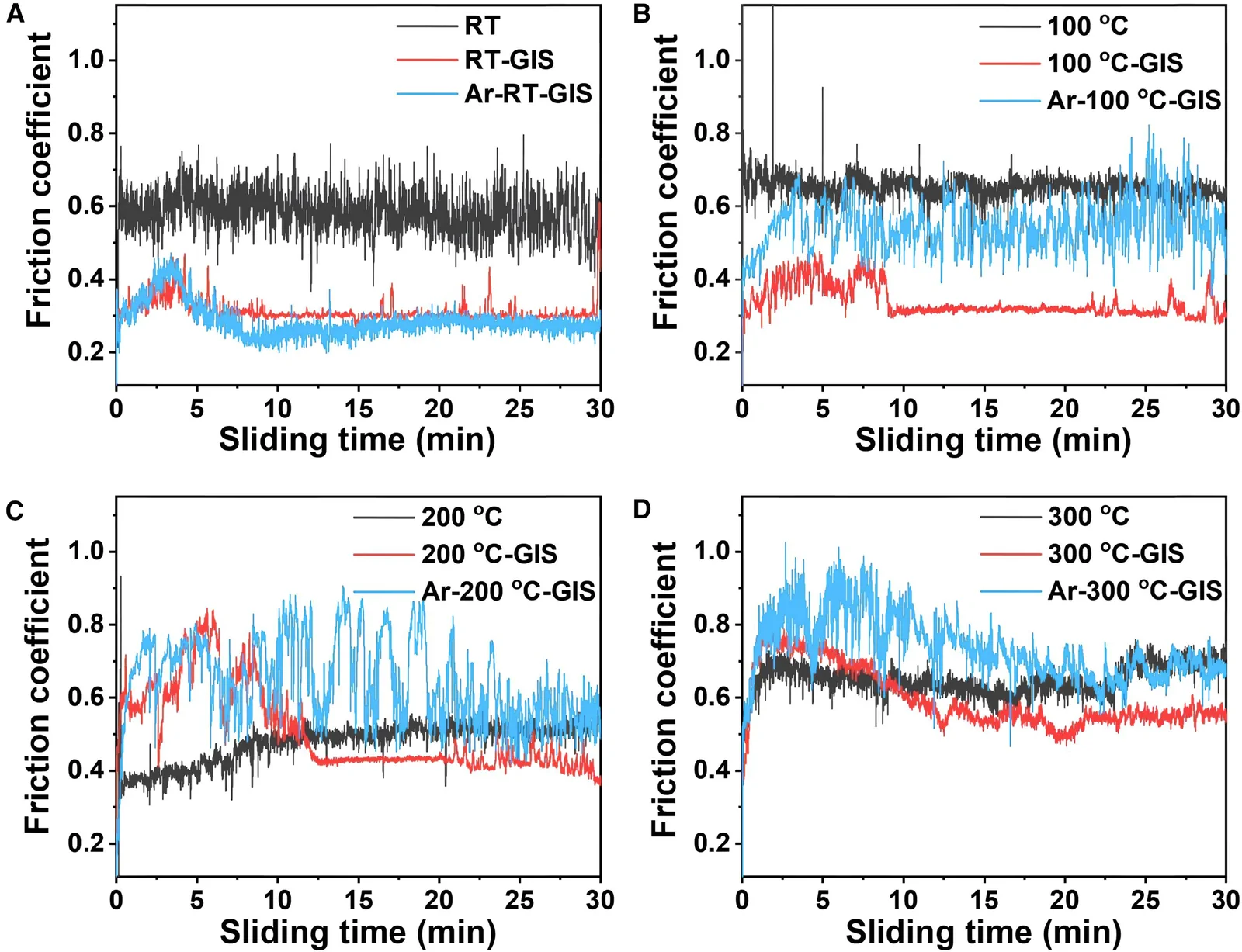
Friction coefficient curves of the Mo/Mo tribopair under dry sliding in air, GIS lubrication in air, and GIS lubrication in argon at different temperatures (A) RT. (B) 100°C. (C) 200°C. (D) 300°C.

**Figure 2:**
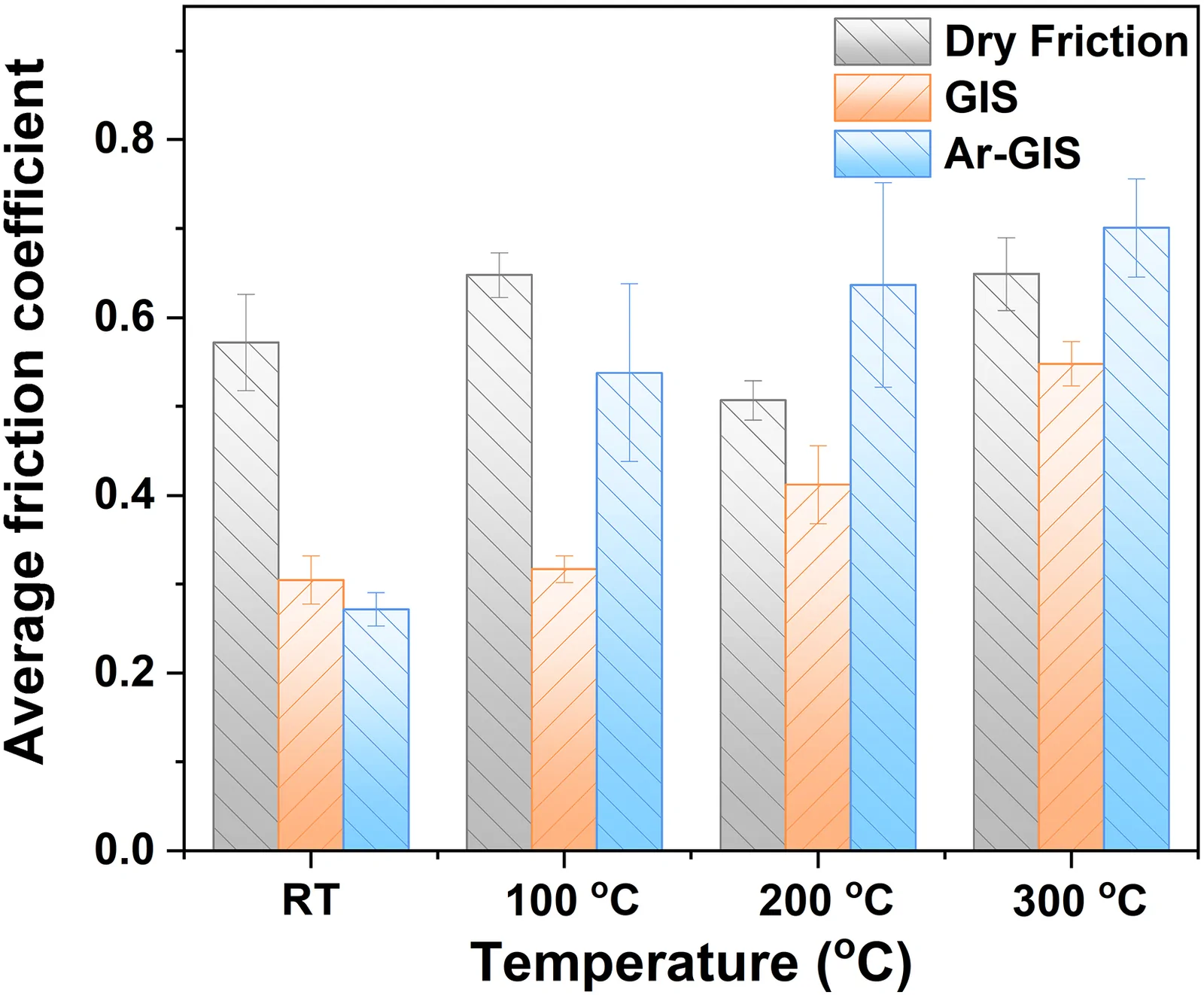
Average friction coefficients of the Mo/Mo sliding pair under dry sliding in air, GIS lubrication in air, and GIS lubrication in argon at different temperatures Data are presented as mean values calculated after excluding the initial running-in stage, with error bars representing the variation in the remaining stage.

After GIS is introduced in air, the friction coefficient curves become much more stable and remain lower than those under dry sliding throughout the RT–300°C range. The average friction coefficients at RT, 100°C, 200°C, and 300°C are 0.31, 0.32, 0.41, and 0.55, respectively. Although the friction coefficient gradually increases with temperature, GIS still significantly reduces interfacial friction over the entire test temperature range, indicating that the spreading/filling effect of the liquid metal and the protection of the tribofilm effectively weaken direct Mo/Mo contact.

In argon, GIS lubrication shows a temperature response that differs markedly from that in air. At RT, the average friction coefficient under GIS lubrication in argon is 0.27, slightly lower than that under GIS lubrication in air, indicating that the intrinsic fluid lubrication and interfacial spreading of the liquid metal still provide effective friction reduction at low temperature. However, as temperature increases, the friction coefficient in argon rises rapidly to 0.54, 0.64, and 0.70 at 100°C, 200°C, and 300°C, respectively, and the friction curves fluctuate more severely. Above 200°C, the friction coefficient under GIS lubrication in argon approaches or exceeds that under dry sliding, suggesting that restricted oxidation suppresses the formation of a protective Ga-O-rich tribofilm and shifts the high-temperature interface toward localized Ga enrichment, delamination, and shear instability.

Overall, the friction coefficient results indicate that GIS in air continuously improves the Mo/Mo friction interface over a wide temperature range, mainly through the synergistic action of the liquid-metal layer and the oxidized tribofilm. Although GIS in argon provides low friction at RT, its lubrication stability deteriorates significantly at elevated temperatures. This phenomenon demonstrates that, for the Mo/Mo-GIS system, moderate oxidation is essential for constructing a stable boundary lubrication film and suppressing direct Ga-Mo interactions.

Figure 3 shows the wear rates of the Mo disk at different temperatures and atmospheres. Under dry sliding in air, the Mo disk undergoes severe wear, but the wear rate decreases with increasing temperature: 135.2, 74.1, 29.8, and 26.7 × 10^−5^ mm^3^/(N·m) at RT, 100°C, 200°C, and 300°C, respectively. This result indicates that high temperature promotes tribo-oxidation of the Mo surface. The resulting oxides are partially compacted and cover the worn surface, reducing the material removal rate. However, the oxide layer is still insufficiently stable, and local cracking and spalling lead to a relatively high friction coefficient. Thus, increasing oxide-layer coverage accounts for the decreasing wear rate, whereas temperature-dependent changes in oxide-layer integrity, cracking, and delamination produce the non-monotonic friction response.

**Figure 3:**
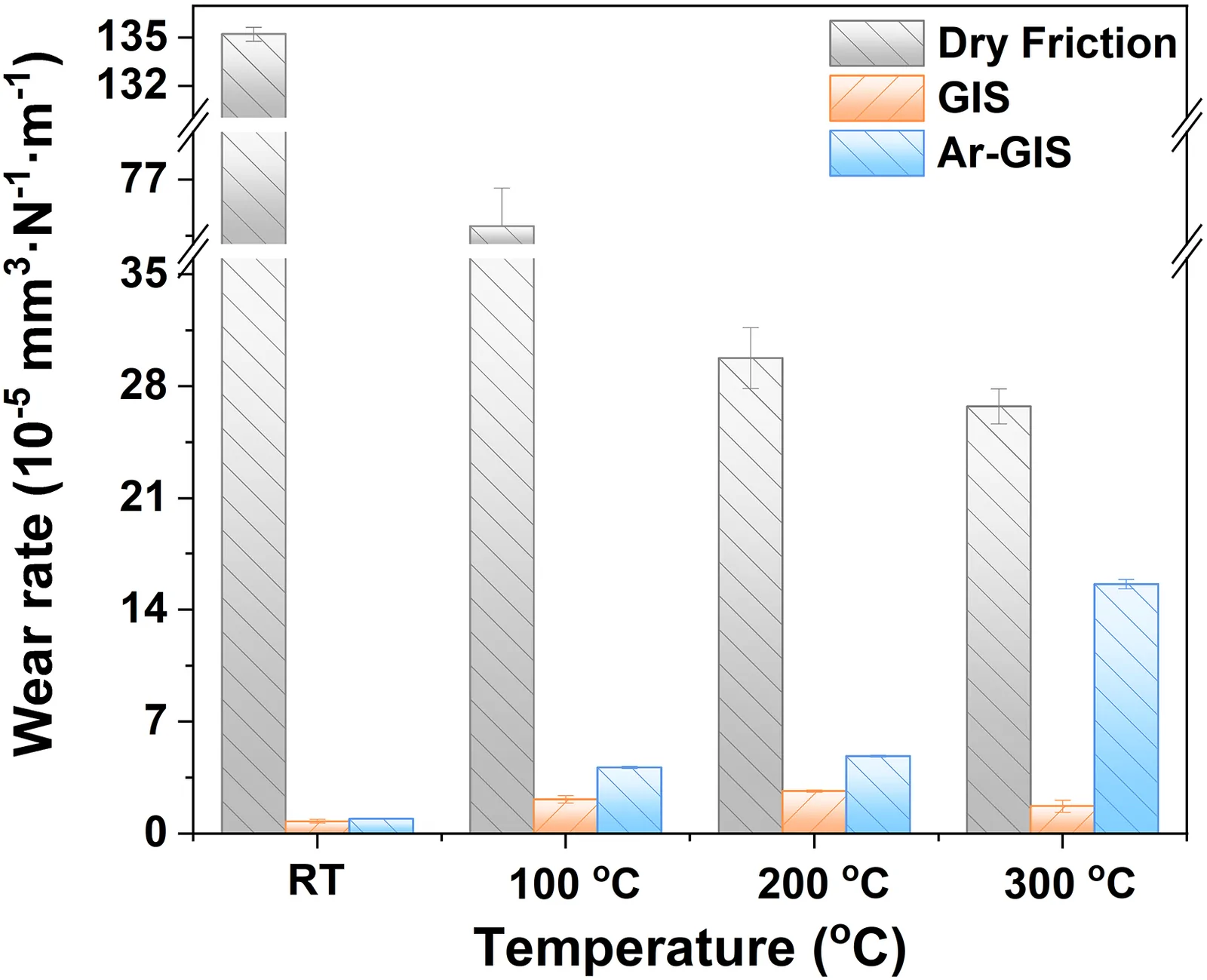
Wear rates of the Mo disk under dry sliding in air, GIS lubrication in air, and GIS lubrication in argon at different temperatures Data are presented as mean ± SD from at least three independent tests.

Under GIS lubrication in air, the wear rate of the Mo disk decreases dramatically to 0.8, 2.1, 2.7, and 1.7 × 10^−5^ mm^3^/(N·m) at RT, 100°C, 200°C, and 300°C, respectively. Compared with dry sliding in air, the wear-rate reduction reaches 90.9%–99.4%, demonstrating the excellent anti-wear capability of GIS for the Mo/Mo self-mated tribopair. Notably, the wear rate under GIS lubrication in air first increases and then decreases with temperature, indicating that temperature simultaneously affects liquid-metal fluidity, Ga oxidation and tribofilm formation, and the load-bearing stability of the tribofilm.

Under GIS lubrication in argon, the wear rate of the Mo disk is consistently higher than that under GIS lubrication in air, with values of 0.9, 4.1, 4.9, and 15.6 × 10^−5^ mm^3^/(N·m) at RT, 100°C, 200°C, and 300°C, respectively. The wear rate increases sharply at 300°C and is approximately nine times that under GIS lubrication in air, indicating that GIS cannot form a sufficiently stable protective film in argon at high temperature. Instead, localized Ga retention and enrichment, reduced near-surface mechanical resistance, and repeated interfacial-layer delamination may intensify surface damage.

The peak in wear rate at 200°C is mainly associated with the unstable state of the tribological interface at this temperature. As observed from the wear morphology, the wear scar is relatively smooth at 100°C, whereas more pronounced layered delamination is observed at 200°C, indicating more frequent damage and detachment of the surface layer during sliding, thereby increasing material removal and resulting in a higher wear rate.

When the temperature is further increased to 300°C, the layered delamination on the wear scar is substantially reduced. Combined with the EDS, X-ray photoelectron spectroscopy (XPS), and cross-sectional characterization results, the higher temperature promotes further oxidation of Ga and the formation of a relatively continuous Ga-O-rich tribofilm, which provides more effective separation and protection of the Mo substrate. Therefore, the higher wear rate at 200°C is mainly associated with the pronounced layered delamination, whereas the enhanced protective effect of the tribofilm at 300°C contributes to suppressing wear.

To further assess the load dependence of the tribological behavior, additional tests were performed at 7 and 13 N under the same conditions. The results showed that the friction coefficient and wear rate varied with applied load, with the 10 N condition exhibiting the lowest values among the tested loads (Figures S3, S4, and S5).

Combining the friction coefficient and wear-rate results, GIS in air provides the most stable wide-temperature lubrication for the Mo/Mo tribopair, maintaining the wear rate of the Mo disk at the 10^–5^ mm^3^/(N·m) level from RT to 300°C. By contrast, GIS lubrication in argon exhibits pronounced temperature sensitivity and a high-temperature failure tendency. These results highlight the important role of atmospheric oxidation in regulating the Mo/Mo-GIS interface and provide a basis for the following analysis of worn morphology and interfacial chemistry.

At RT, GIS exhibited comparable lubrication performance under air and Ar atmospheres. The differences in friction and wear between the two atmospheres were relatively small and within experimental uncertainty. The slightly higher friction but lower wear tendency in air may be related to the formation of a more stable interfacial layer, which increases interfacial shear resistance while improving surface protection.

### Worn morphology and interfacial chemistry of the Mo disk

Figure 4 shows the three-dimensional wear scars and SEM morphologies of the Mo disks at RT under the three friction conditions, and Table 1 lists the EDS results from representative regions of the worn surfaces. Under dry sliding, the Mo disk exhibits a wide and deep wear scar with obvious tearing, grooves, plastic deformation, and local spalling along the sliding direction, indicating severe adhesive and abrasive wear. The EDS results show the presence of O on the worn surface, suggesting mild tribo-oxidation induced by frictional heating. After GIS addition, the wear scar in air becomes much shallower and the surface becomes smoother, with no obvious large-scale tearing or spalling. The detection of Ga, In, and Sn on the worn surface confirms that the liquid metal participates in interfacial film formation. Compared with air, GIS lubrication in argon produces a slightly rougher wear scar with mild spalling and plastic deformation, and the surface oxygen content decreases, indicating that insufficient oxide-film formation weakens the stable boundary lubrication ability of GIS on Mo.

**Figure 4:**
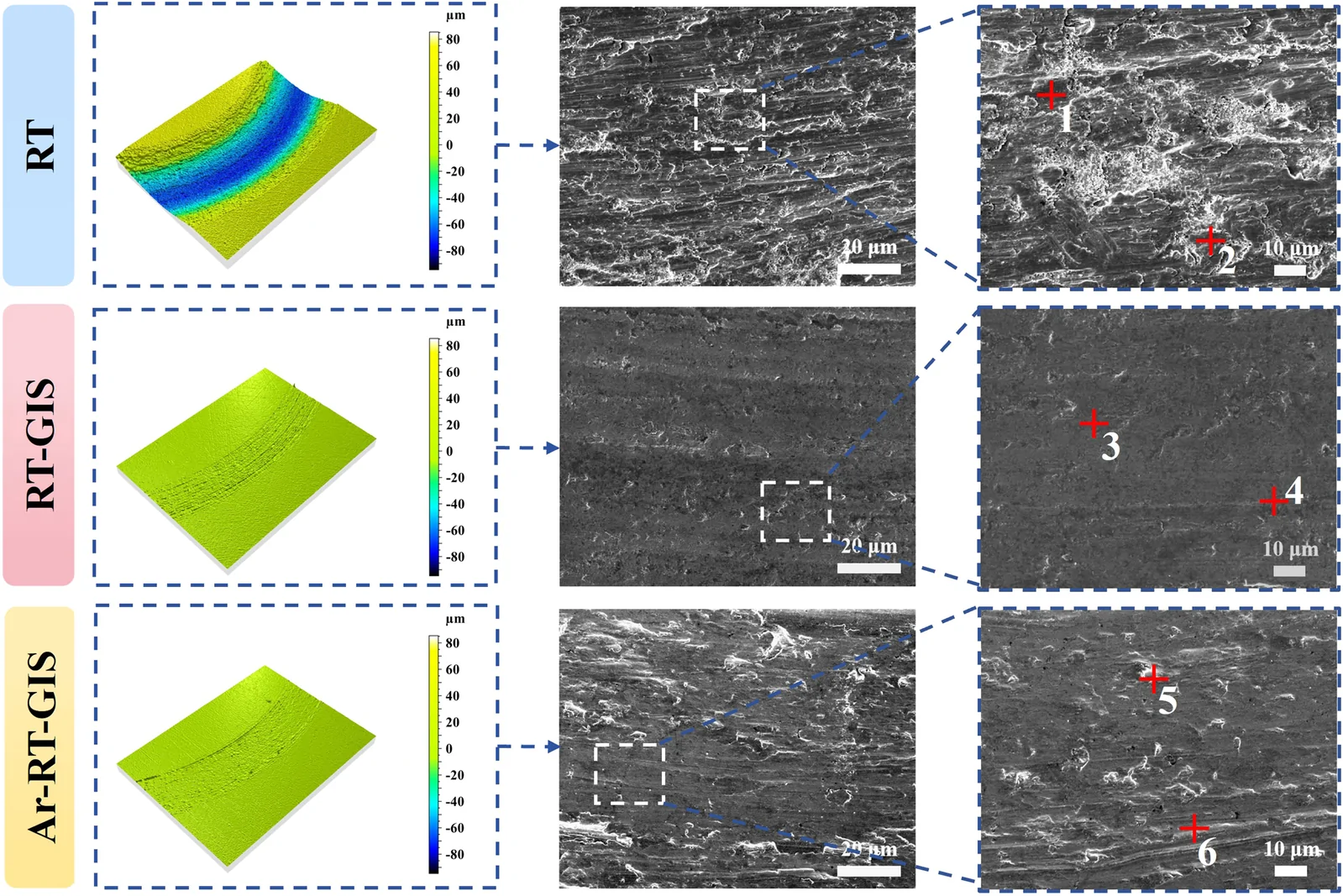
Three-dimensional morphology and SEM images of the worn surface of the Mo disk at RT under dry sliding in air, GIS lubrication in air, and GIS lubrication in argon Scale bars: 20 μm in the SEM overview images and 10 μm in the enlarged SEM images.

**Table 1:** Chemical compositions of representative regions on the worn surface of the Mo disk under different friction conditions at RT

Region	Composition (atom %)	Region	Composition (atom %)
1	Mo_76.12_O_23.88_	4	Mo_65.81_O_24.98_Ga_7.33_In_1.05_Sn_0.83_
2	Mo_72.76_O_27.24_	5	Mo_79.88_O_11.78_Ga_6.75_In_0.55_Sn_1.04_
3	Mo_70.19_O_17.21_Ga_11.14_In_0.39_Sn_1.07_	6	Mo_77.17_O_18.50_Ga_2.91_In_0.32_Sn_1.10_

Figure 5 shows the worn morphology of the Mo disk at 100°C, and Table 2 summarizes the compositional data for representative regions of the worn surface. Under dry sliding in air, the wear-scar depth decreases compared with RT, and tearing along the sliding direction becomes less pronounced, although plastic deformation and local spalling are still observed. The EDS results show an increased O content, indicating that elevated temperature promotes surface oxidation of Mo. The oxides provide a certain load-bearing and separating effect at the friction interface,^31^ so the wear rate is lower than that at RT under dry sliding. Under GIS lubrication in air, the worn surface remains generally smooth, but local spalling begins to appear, suggesting that temperature-induced changes in GIS properties and intensified interfacial reactions reduce the stability of the lubricating film. Under GIS lubrication in argon, local spalling becomes more pronounced and the Ga signal increases, indicating enhanced physical retention and localized enrichment of GIS when oxidation is restricted. This interfacial instability likely contributes to the increased friction coefficient and enhanced fluctuation.

**Figure 5:**
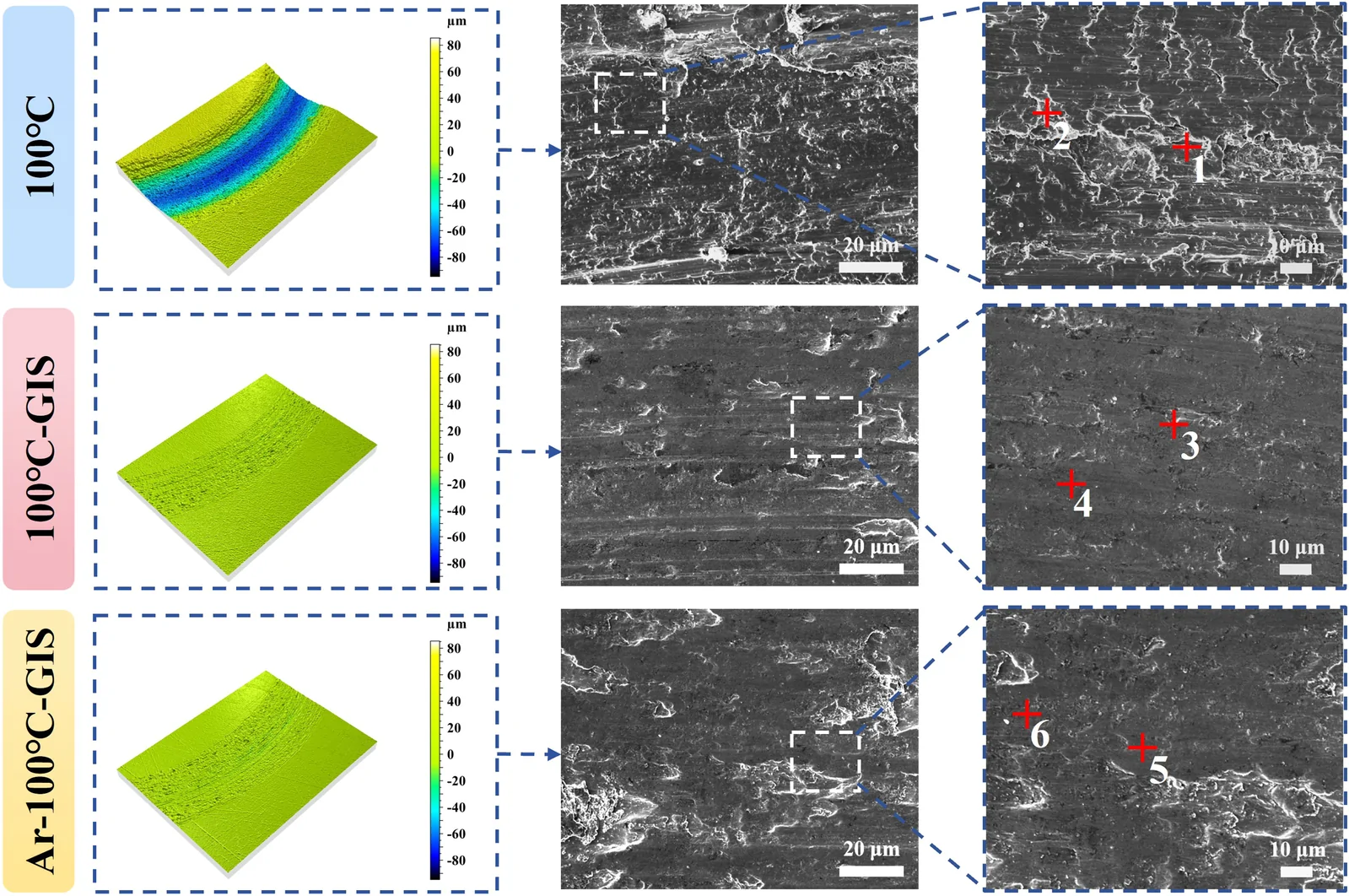
Three-dimensional morphology and SEM images of the worn surface of the Mo disk at 100°C under dry sliding in air, GIS lubrication in air, and GIS lubrication in argon Scale bars: 20 μm in the SEM overview images and 10 μm in the enlarged SEM images.

**Table 2:** Chemical compositions of representative regions on the worn surface of the Mo disk under different friction conditions at 100°C

Region	Composition (atom %)	Region	Composition (atom %)
1	Mo_74.66_O_25.34_	4	Mo_48.66_O_27.31_Ga_21.56_In_1.48_Sn_0.99_
2	Mo_63.16_O_36.84_	5	Mo_66.51_O_17.77_Ga_13.37_In_1.20_Sn_1.15_
3	Mo_68.16_O_18.20_Ga_10.55_In_1.84_Sn_1.25_	6	Mo_46.29_O_18.31_Ga_33.09_In_1.19_Sn_1.13_

Figure 6 shows the worn morphology of the Mo disk at 200°C, with the compositional data for representative regions of the worn surface summarized in Table 3. Under dry sliding, the wear-scar depth further decreases and the oxygen content on the worn surface continues to increase, indicating that a stronger tribo-oxide layer suppresses substrate removal to some extent and reduces spalling and plastic deformation compared with lower temperatures. Under GIS lubrication in air, lamellar spalling appears on the worn surface, and the wear rate is higher than those at RT and 100°C, indicating that enhanced liquid-metal fluidity, Ga oxidation, and interfacial reactions jointly modify the tribofilm structure at this temperature. Under GIS lubrication in argon, spalling is markedly intensified and plastic deformation similar to that under dry sliding appears. EDS results show that the Ga content exceeds 50 atom %, confirming that a large amount of GIS adheres to the worn surface. Repeated formation and removal of this Ga-enriched interfacial layer under frictional shear likely contribute to severe friction fluctuation and increased wear in argon.

**Figure 6:**
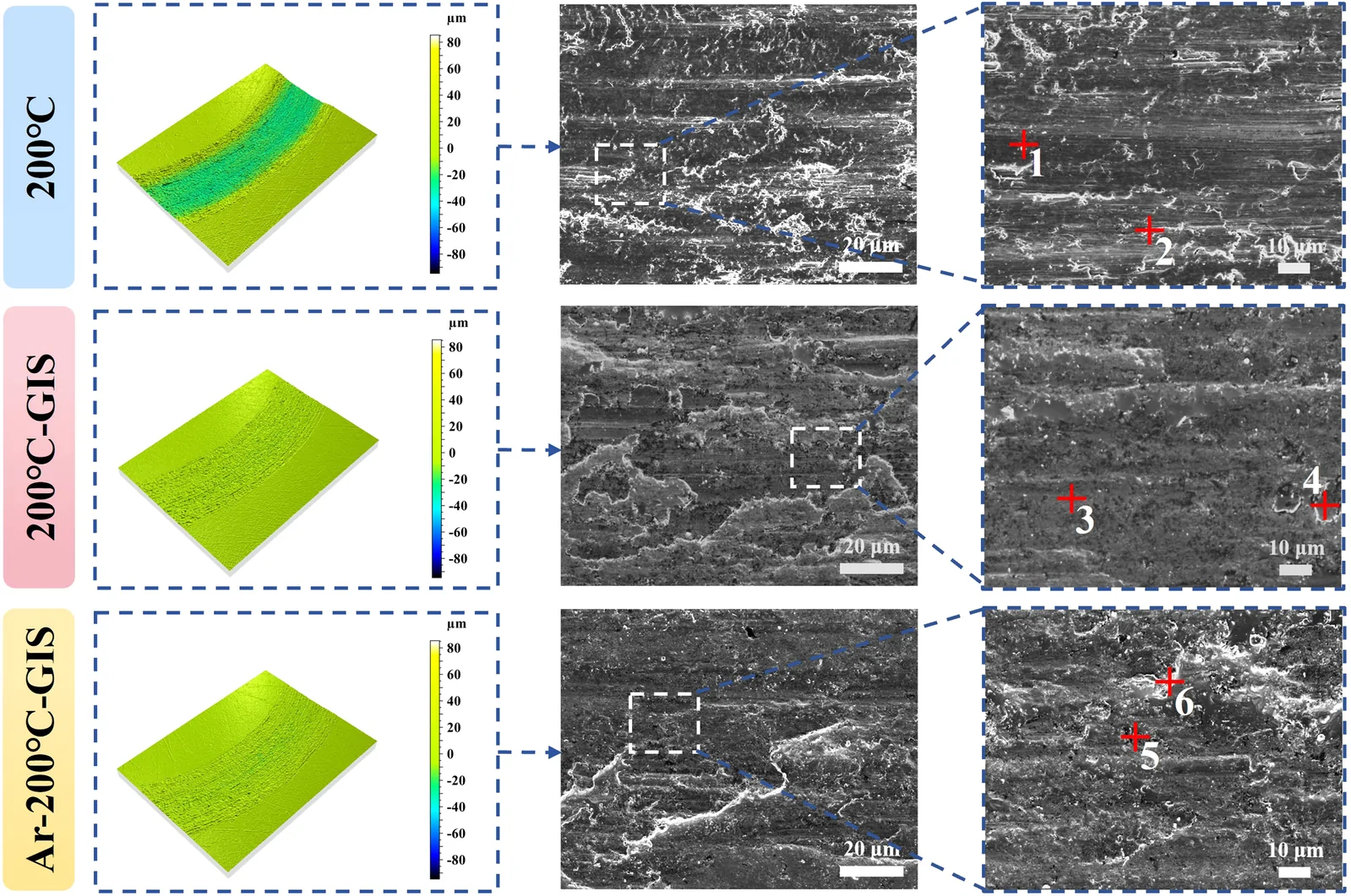
Three-dimensional morphology and SEM images of the worn surface of the Mo disk at 200°C under dry sliding in air, GIS lubrication in air, and GIS lubrication in argon Scale bars: 20 μm in the SEM overview images and 10 μm in the enlarged SEM images.

**Table 3:** Chemical compositions of representative regions on the worn surface of the Mo disk under different friction conditions at 200°C

Region	Composition (atom %)	Region	Composition (atom %)
1	Mo_69.60_O_30.40_	4	Mo_43.90_O_31.48_Ga_22.74_In_0.81_Sn_1.08_
2	Mo_60.96_O_39.04_	5	Mo_21.30_O_11.08_Ga_53.93_In_8.99_Sn_4.70_
3	Mo_48.46_O_20.77_Ga_28.63_In_1.22_Sn_0.93_	6	Mo_12.91_O_17.11_Ga_58.45_In_7.49_Sn_4.03_

Figure 7 shows the worn morphology of the Mo disk at 300°C, with the corresponding regional compositional data summarized in Table 4. Under dry sliding, the wear-scar depth further decreases and the O content reaches a high level, indicating that high temperature promotes the formation of a more continuous tribo-oxide layer and further reduces the wear rate. However, local spalling traces perpendicular to the sliding direction suggest that fatigue cracking still occurs in the oxide layer. Under GIS lubrication in air, the Mo disk surface is relatively smooth, with only a few small spalling defects and fine abrasive-wear traces. Its wear rate is lower than those at 100°C and 200°C, demonstrating that the Ga-O-rich tribofilm developed at high temperature, together with the liquid-metal layer, provides strong interfacial protection. In contrast, under GIS lubrication in argon, obvious spalling and plastic deformation appear on the worn surface. Localized Ga retention and repeated delamination of the unstable interfacial layer ultimately cause a significant increase in wear rate at 300°C.

**Figure 7:**
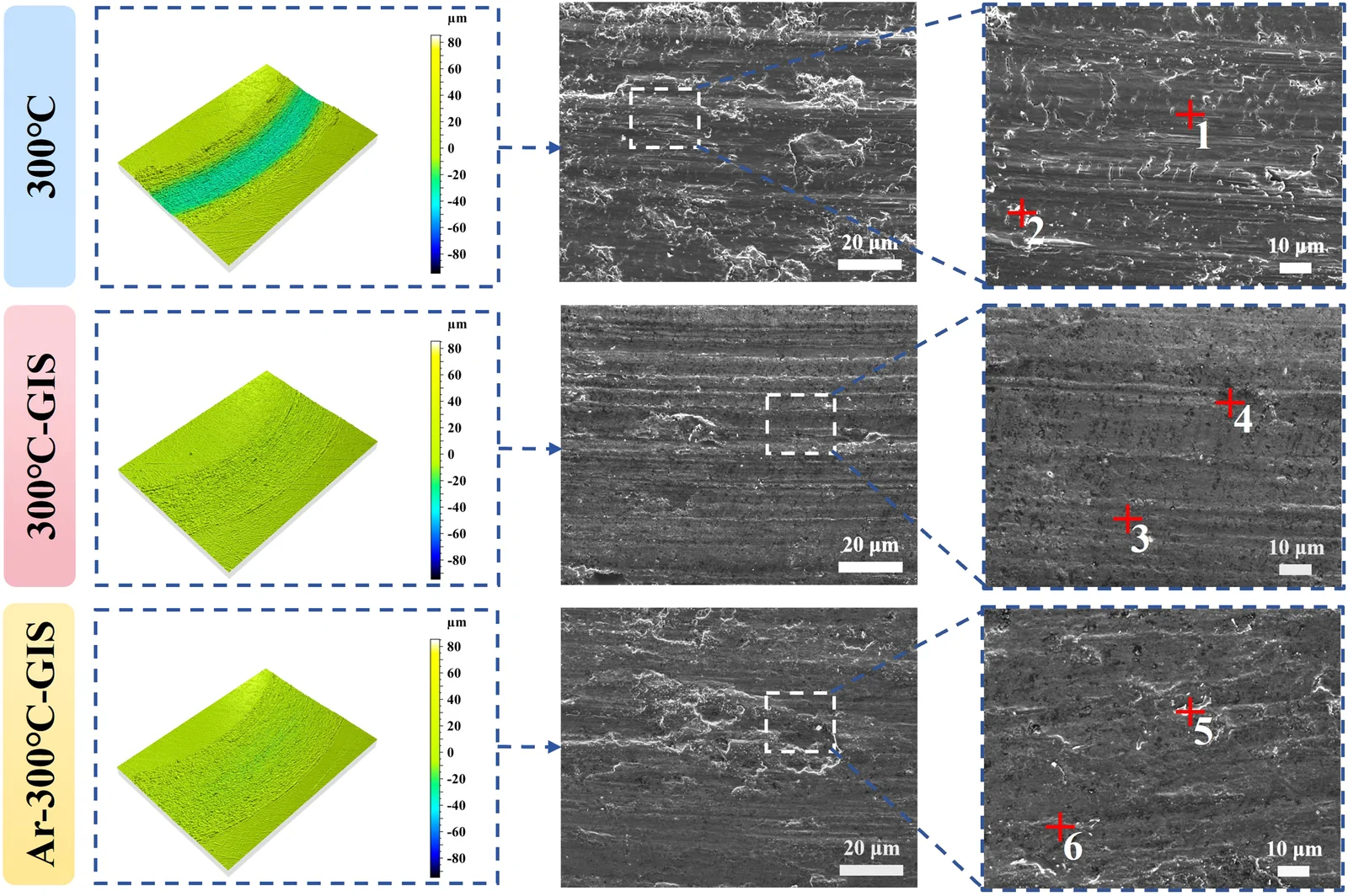
Three-dimensional morphology and SEM images of the worn surface of the Mo disk at 300°C under dry sliding in air, GIS lubrication in air, and GIS lubrication in argon Scale bars: 20 μm in the SEM overview images and 10 μm in the enlarged SEM images.

**Table 4:** Chemical compositions of representative regions on the worn surface of the Mo disk under different friction conditions at 300°C

Region	Composition (atom %)	Region	Composition (atom %)
1	Mo_60.16_O_39.84_	4	Mo_51.54_O_32.71_Ga_11.97_In_1.98_Sn_1.79_
2	Mo_55.13_O_44.87_	5	Mo_13.52_O_7.00_Ga_70.83_In_5.46_Sn_3.19_
3	Mo_54.02_O_26.24_Ga_17.55_In_1.23_Sn_0.95_	6	Mo_47.64_O_18.66_Ga_30.56_In_1.93_Sn_1.22_

### Worn morphology of the Mo pin

Figure 8 shows the SEM morphologies of the worn surfaces of the Mo pins after sliding at RT under different friction conditions. Under dry sliding, GIS lubrication in air, and GIS lubrication in argon, the wear lengths along the sliding direction are 1,782.81, 708.43, and 771.54 μm, respectively, showing a significant reduction from dry sliding to GIS lubrication and a slight increase from GIS lubrication in air to GIS lubrication in argon. Under dry sliding, numerous spalling pits and grooves are observed on the Mo pin surface, indicating severe adhesive and abrasive wear at the contact interface. After GIS lubrication in air, the surface becomes much smoother, with only slight spalling. Under GIS lubrication in argon, mild spalling pits and grooves are observed. This result is consistent with the wear rate and worn morphology of the Mo disk, further confirming that GIS significantly reduces material damage on both sides of the Mo/Mo contact pair.

**Figure 8:**
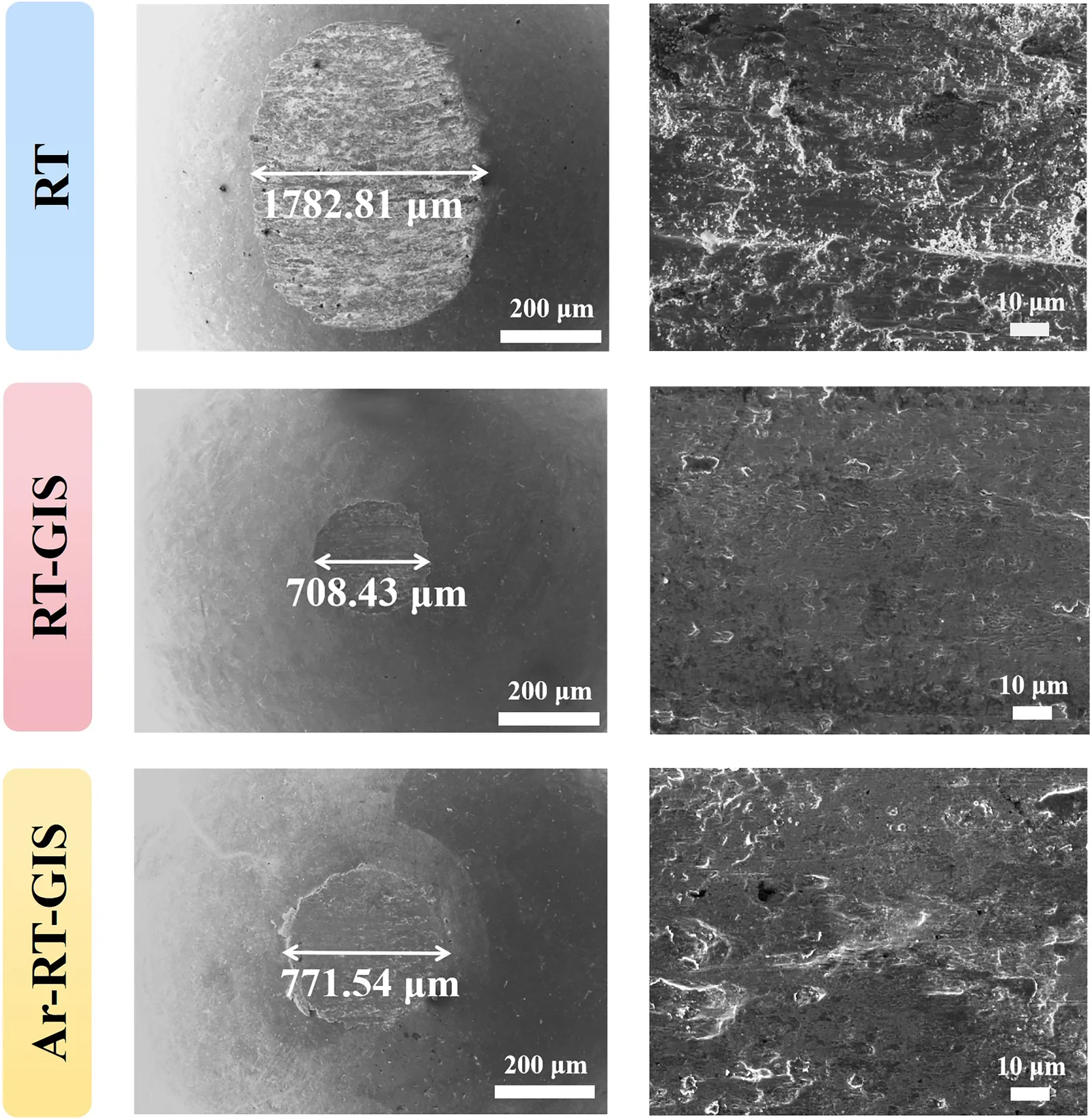
SEM images of the worn surface of the Mo pin at RT under dry sliding in air, GIS lubrication in air, and GIS lubrication in argon Scale bars: 200 μm in the low-magnification images and 10 μm in the high-magnification images.

Figure 9 shows the worn surface morphology of the Mo pin at 100°C. Under dry sliding, GIS lubrication in air, and GIS lubrication in argon, the wear lengths along the sliding direction are 1,339.94, 995.19, and 1,058.63 μm, respectively. Compared with RT, spalling and grooves on the Mo pin under dry sliding are reduced, indicating that tribo-oxidation promoted by elevated temperature suppresses wear to some extent. Both GIS lubrication conditions significantly reduce the wear size at the pin end. The surface under GIS lubrication in air is smoother, whereas that under GIS lubrication in argon is slightly rougher and shows residual GIS deposits, suggesting poorer liquid-metal film stability in the inert atmosphere.

**Figure 9:**
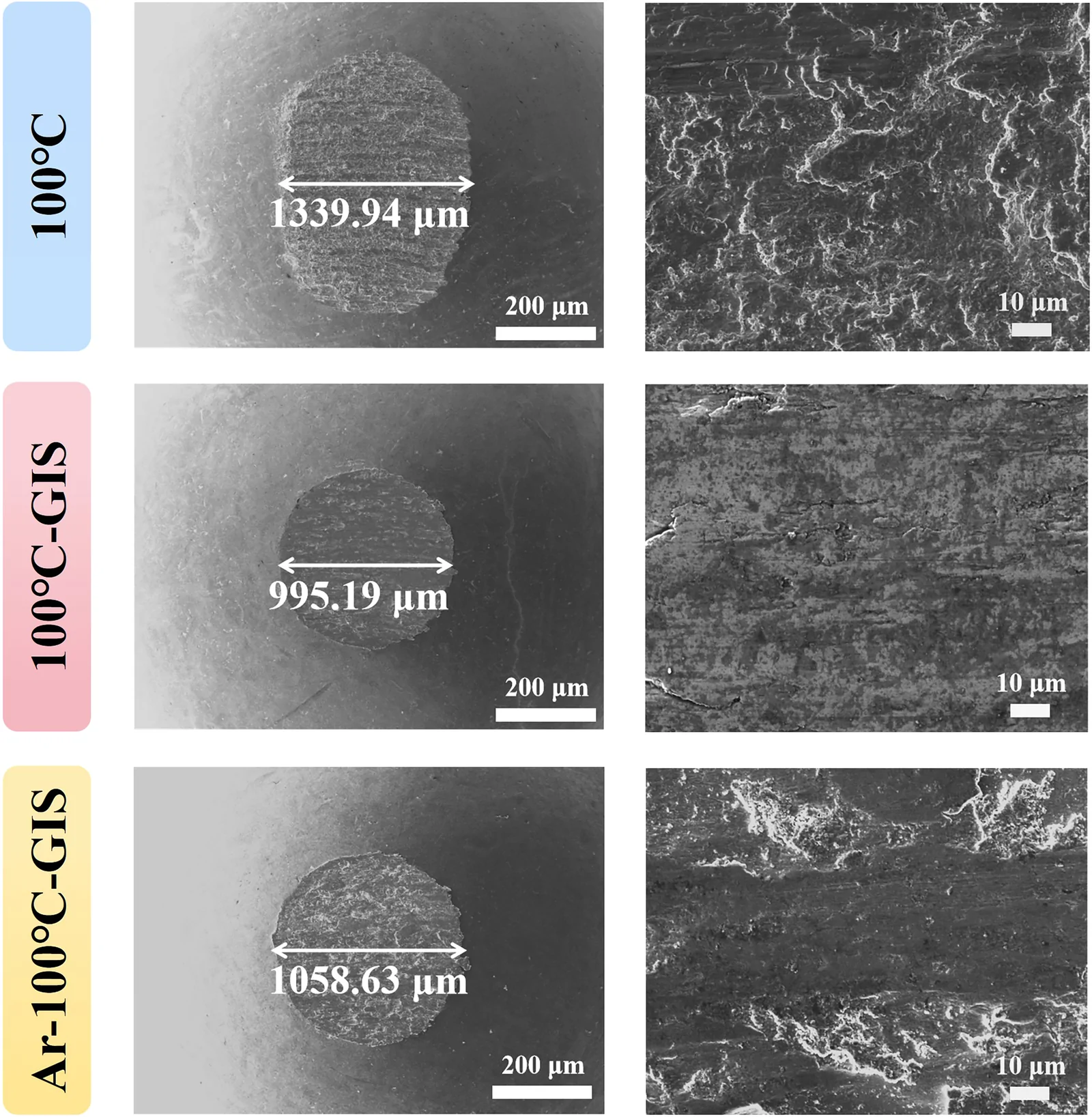
SEM images of the worn surface of the Mo pin at 100°C under dry sliding in air, GIS lubrication in air, and GIS lubrication in argon Scale bars: 200 μm in the low-magnification images and 10 μm in the high-magnification images.

Figure 10 shows the worn surface morphology of the Mo pin at 200°C. Under dry sliding, GIS lubrication in air, and GIS lubrication in argon, the wear lengths along the sliding direction are 1,271.91, 1,011.41, and 1,105.36 μm, respectively. The trend again indicates that GIS lubrication reduces wear, whereas argon weakens the lubrication effect. Spalling and grooves remain on the dry-sliding surface, but the damage is less severe than at lower temperatures. Under GIS lubrication in air, the surface is relatively smooth with only slight local spalling. Under GIS lubrication in argon, spalling becomes more severe and is accompanied by more GIS adhesion, indicating that strong adhesion between the liquid metal and Mo surface at high temperature in argon can induce unstable delamination.

**Figure 10:**
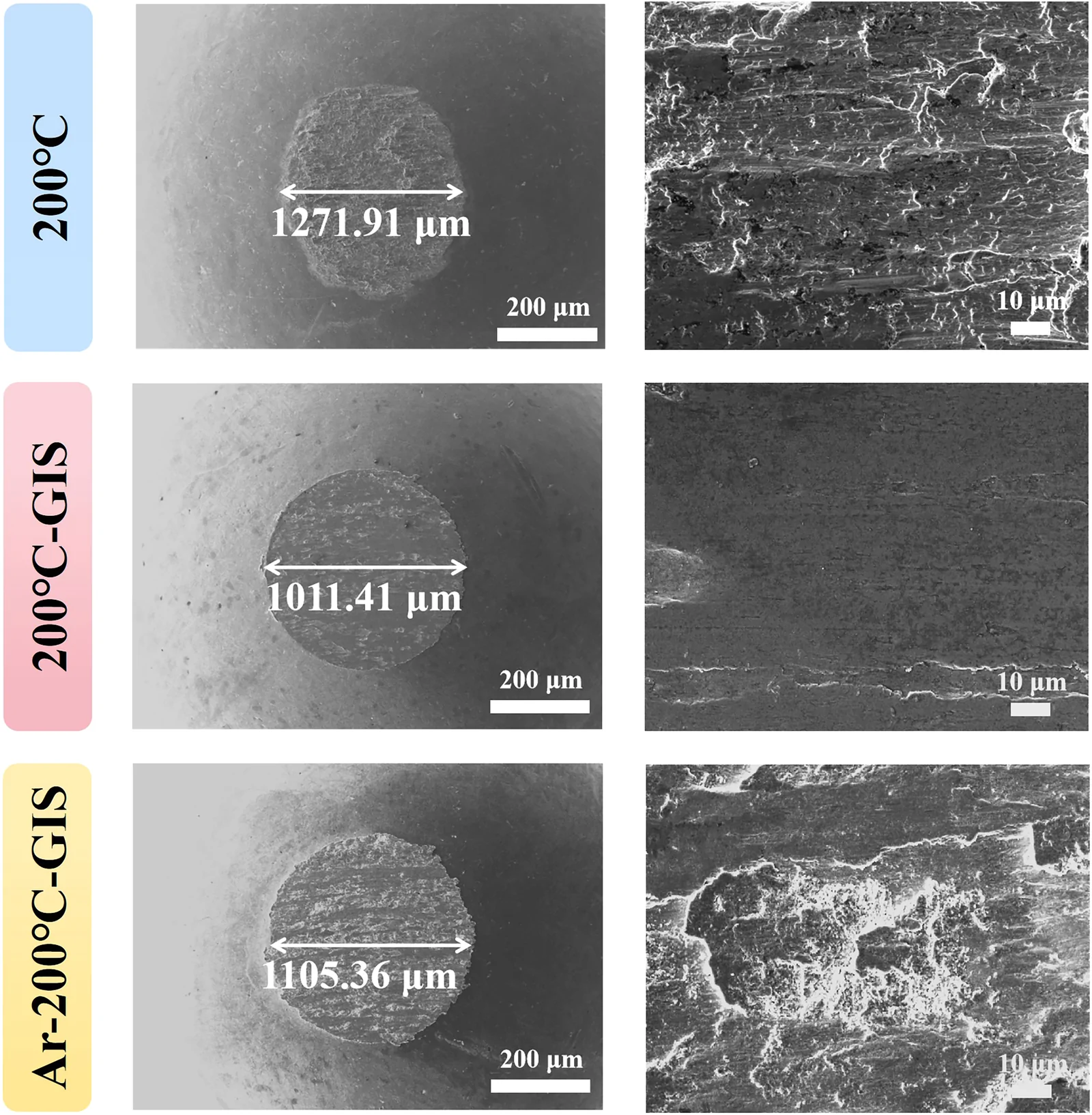
SEM images of the worn surface of the Mo pin at 200°C under dry sliding in air, GIS lubrication in air, and GIS lubrication in argon Scale bars: 200 μm in the low-magnification images and 10 μm in the high-magnification images.

Figure 11 shows the worn surface morphology of the Mo pin at 300°C. Under dry sliding, GIS lubrication in air, and GIS lubrication in argon, the wear lengths along the sliding direction are 1,201.87, 882.97, and 1,152.93 μm, respectively. Under GIS lubrication in air, the pin end shows the mildest wear and a relatively smooth surface, indicating that the Ga-O-rich composite film formed in air also protects the Mo pin. Under GIS lubrication in argon, surface spalling is markedly intensified, and the wear length approaches that under dry sliding, indicating insufficient high-temperature film stability together with Ga-associated interfacial weakening.

**Figure 11:**
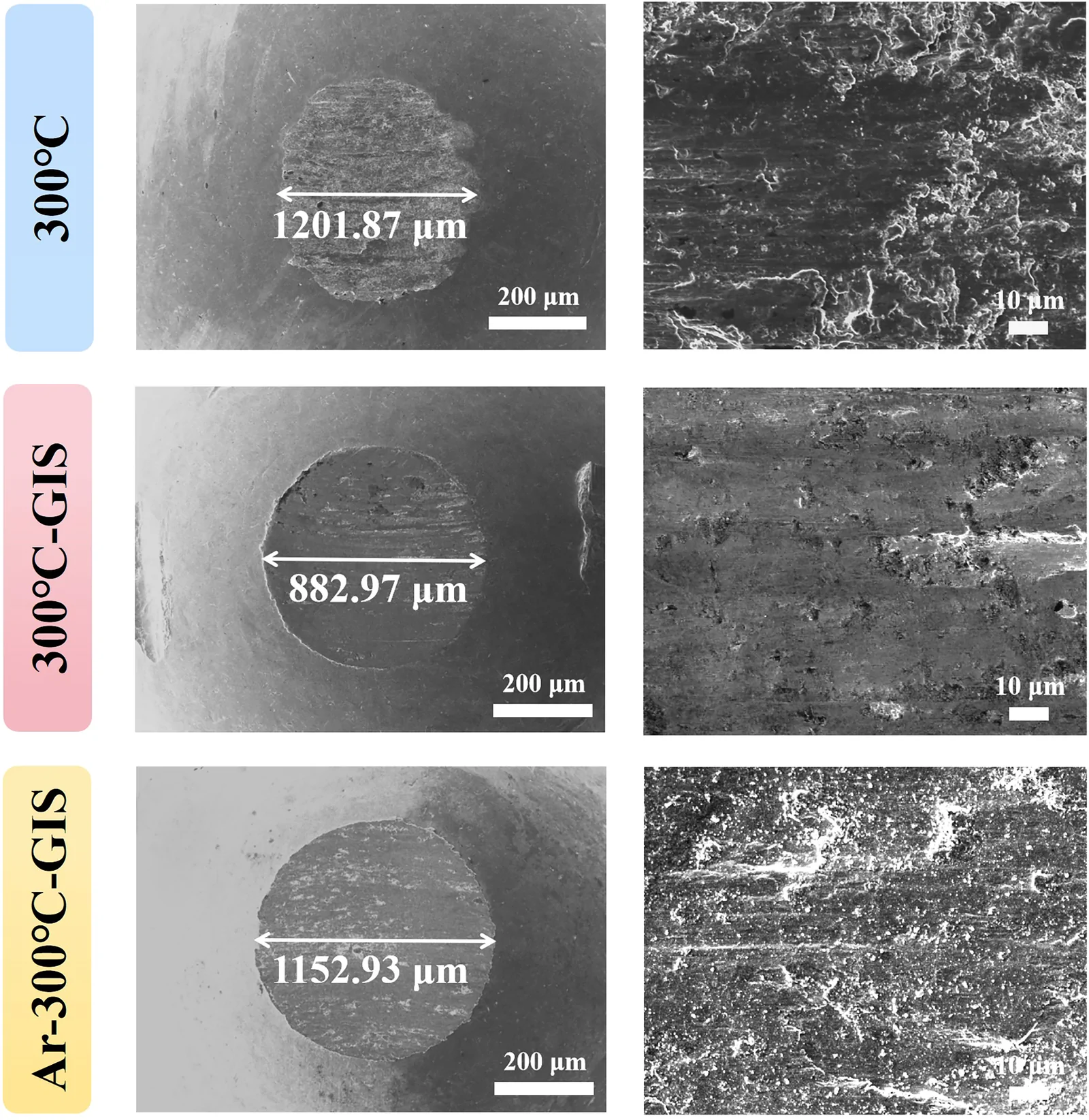
SEM images of the worn surface of the Mo pin at 300°C under dry sliding in air, GIS lubrication in air, and GIS lubrication in argon Scale bars: 200 μm in the low-magnification images and 10 μm in the high-magnification images.

## Discussion

Under dry sliding in air, the wear behavior of the Mo/Mo tribopair is mainly controlled by the formation and spalling of a tribo-oxide layer. As temperature increases, the O content on the Mo disk surface increases from 23–27 atom % to 39–45 atom %, indicating that high temperature strongly promotes tribo-oxidation of Mo. The resulting Mo oxides are repeatedly compacted under contact stress and can form a tribo-oxide layer with a certain load-bearing capability. This layer reduces direct contact between the Mo substrate and the counterface, suppressing adhesive wear and deep plowing.^32^^,^^33^^,^^34^ Consequently, the wear rate under dry sliding gradually decreases with temperature. The friction coefficient, however, varies with the continuity and damage state of the oxide layer. At 100°C, the layer remains incomplete, and the worn surface exhibits plastic deformation and local delamination, resulting in relatively high interfacial shear resistance. At 200°C, the oxide layer becomes more continuous, while plastic deformation and delamination are reduced, leading to a lower friction coefficient. At 300°C, enhanced oxidation still limits material loss, but pronounced cracking and local delamination of the oxide layer increase interfacial shear resistance again. Thus, the increasing protective coverage of the oxide layer accounts for the decreasing wear rate, whereas temperature-dependent changes in its integrity and delamination behavior produce the non-monotonic friction response.

Compared with dry sliding, GIS rapidly spreads and enters the Mo/Mo contact interface at RT. The low-shear characteristics and filling effect of the liquid-metal layer reduce the real contact area,^35^ decreasing the friction coefficient from approximately 0.57 under dry sliding to 0.27–0.31 and the wear rate of the Mo disk from 135.2 × 10^−5^ mm^3^/(N·m) to approximately 0.8–0.9 × 10^−5^ mm^3^/(N·m). However, in argon, the lubrication performance of GIS deteriorates significantly as temperature increases. This can be attributed to the following factors. First, high temperature lowers the viscosity of GIS and promotes its spreading and localized retention on the Mo surface.^36^^,^^37^ Under restricted oxidation, Ga can also accumulate within surface defects and mechanically damaged near-surface regions.^25^ Cross-sectional SEM observations after sliding at 300°C in Ar reveal a relatively shallow deformed and damaged region beneath the worn surface, with localized morphological variations in the near-surface area (Figure S6). Cross-sectional EDS mapping further confirms that the Ga signal is concentrated near the worn surface and locally extends into the near-surface damaged region of the Mo disk (Figure S7). This observation suggests localized Ga ingress or retention within the mechanically damaged subsurface region, rather than confinement of Ga exclusively to the outermost surface. Ga exhibits a relatively continuous distribution along the worn surface, indicating relatively continuous coverage of the Mo substrate within the examined region. Nanoindentation further shows lower hardness and reduced *H*_*IT*_*/E*_*r*_ and *H*_*IT*_^*3*^*/E*_*r*_^*2*^ values in the Ga-affected region than in the reference Mo substrate (Figure S8; Table S1). These representative measurements indicate lower hardness, reduced elastic-strain tolerance, and substantially decreased resistance to plastic deformation in the Ga-affected region. Therefore, Ga enrichment is considered a factor that may promote interfacial weakening, subsurface cracking, and layered delamination. Together with temperature-dependent changes in the liquid structure of GIS,^16^^,^^28^^,^^38^ these effects contribute to the increased friction and wear at 300°C in Ar.

As shown in Figure S9, the cross-sectional SEM image of the GIS-lubricated Mo wear scar tested at 300°C in air reveals the interface between the surface layer and the underlying Mo substrate. The corresponding EDS elemental mapping in Figure S10 shows that Ga is predominantly localized in the near-surface region and exhibits a relatively continuous distribution along the worn surface, whereas Mo remains mainly concentrated in the substrate. These results suggest that the Ga-containing surface layer is relatively continuously distributed over the Mo substrate within the examined region.

Figure 12 shows the Ga 2p and Mo 3d XPS spectra of the worn surface of the Mo disk after GIS lubrication in air at different temperatures. In the Ga 2p spectra, the Ga^3+^ peak remains at approximately 1118.01 eV, while the metallic Ga^0^ peak appears at 1116.68–1116.74 eV, indicating the coexistence of oxidized Ga and metallic Ga on the worn surface.^39^ The dominant oxidized Ga component indicates preferential Ga oxidation in air. Because XPS provides chemical-state information but cannot resolve the detailed structure or morphology of the interfacial layer, it is described as a Ga-O-rich tribofilm. As temperature increases, the overall content of Ga oxides increases, consistent with the increasing O content identified by EDS. The Mo 3d spectra mainly exhibit a doublet at approximately 227.98–228.01 eV and 231.14–231.19 eV, with a spin-orbit splitting of about 3.1 eV, which is characteristic of metallic Mo.^40^^,^^41^ No clear evidence of Ga-Mo reaction or Mo-Ga compound formation was detected up to 300°C. These Ga-O species may arise from native oxidation, thermal oxidation, friction-assisted oxidation, or their combined effects.^42^ Figure S11 presents the high-resolution XPS spectra of Ga 2p and Mo 3d obtained from the GIS-lubricated Mo wear scar tested at 300°C in Ar. Both Ga^0^ and Ga^3+^ were detected, with a Ga^3+^/Ga^0^ ratio of 2.86, lower than that under air (3.30), indicating that Ga oxidation was partially suppressed under Ar. The Mo 3d spectrum retained the characteristic metallic Mo peaks.

**Figure 12:**
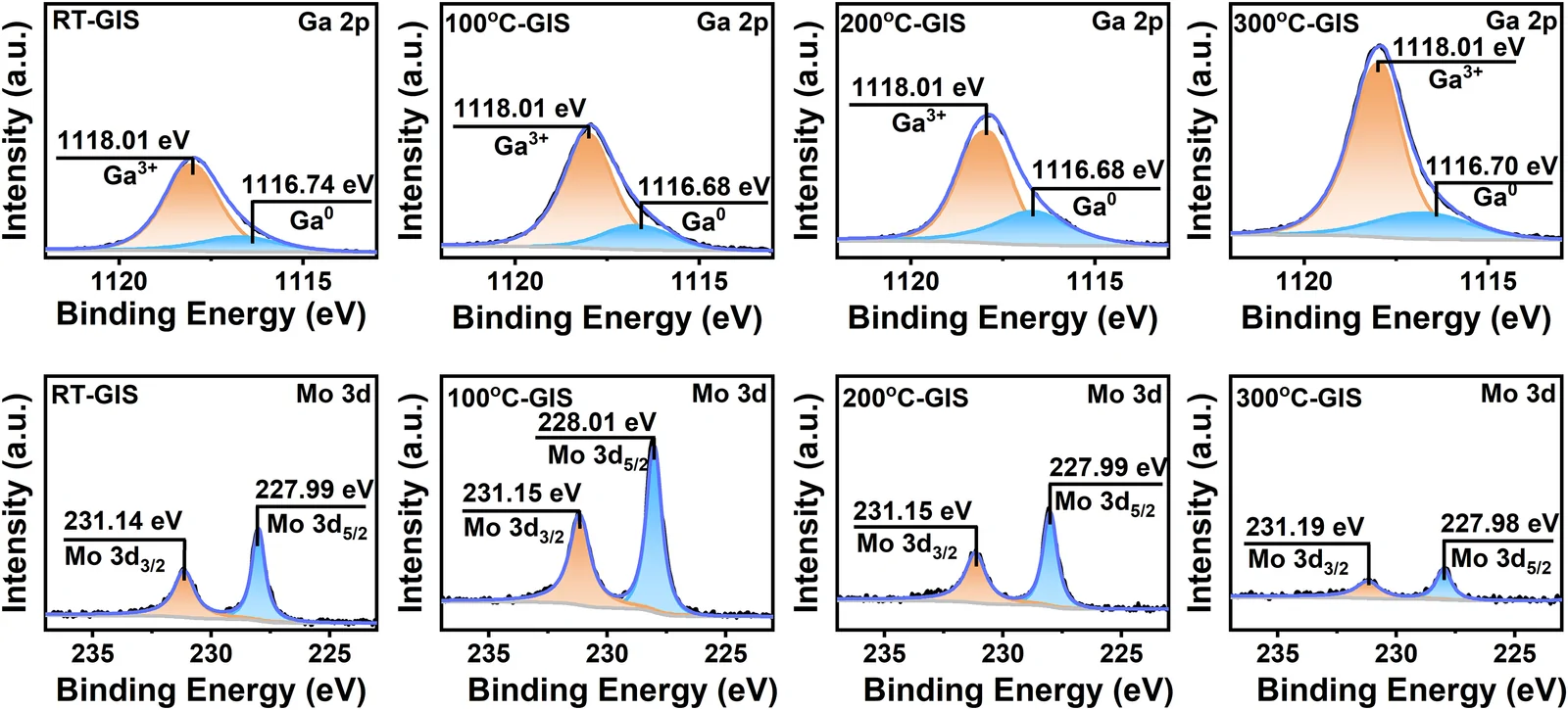
XPS spectra of the worn surface of the Mo disk after GIS lubrication in air at different temperatures

Therefore, the efficient lubrication of the Mo/Mo tribopair by GIS in air can be attributed to a liquid-metal/Ga-O-rich composite lubrication mechanism. During sliding, GIS first fills surface asperities through its good fluidity and wettability, reducing direct Mo/Mo contact. Oxygen in air then promotes preferential Ga oxidation and the development of a Ga-O-rich tribofilm. Under frictional shear, the interfacial layer may undergo repeated formation, local rupture or delamination, spreading, and replenishment by the liquid metal.^43^ The film also limits direct exposure of the Mo surface to active retained Ga,^44^ thereby reducing Ga-associated near-surface damage and large-scale lamellar spalling. As a result, GIS lubrication in air maintains a low wear rate of 0.8–2.7 × 10^−5^ mm^3^/(N·m) over the RT–300°C range. The wear rate of the Mo disk lubricated by GIS increases from RT to 200°C and then decreases at 300°C. The EDS results reveal a markedly higher oxygen content on the worn surface at 300°C than at lower temperatures, demonstrating that the elevated temperature promotes Ga oxidation at the sliding interface. Together with the oxidized Ga species identified by XPS, this finding indicates that more extensive Ga oxidation at 300°C favors the formation of a Ga-O-rich tribofilm with greater coverage and continuity. At 200°C, interfacial oxidation is less extensive, and the resulting tribofilm is comparatively less continuous and stable, providing weaker wear protection. Therefore, the reduced wear rate at 300°C is attributed mainly to enhanced Ga oxidation and the more effective interfacial separation provided by the resulting Ga-O-rich tribofilm.

In argon, restricted oxidation results in localized Ga enrichment and a comparatively discontinuous interfacial layer, promoting delamination and shear instability and thereby degrading the lubrication performance. The atmosphere- and temperature-regulated lubrication mechanism of GIS for the Mo/Mo tribopair is shown in Figure 13. In air, increasing the temperature from RT to 300°C progressively promotes Ga oxidation, resulting in a more continuous and stable Ga-O-rich tribofilm at the sliding interface. Under Ar, oxidation is restricted, and the interface is characterized mainly by localized Ga enrichment and a comparatively discontinuous interfacial layer.

(1) Under dry sliding in air, severe friction and wear occur in the Mo/Mo self-mated tribopair. As temperature increases from RT to 300°C, tribo-oxidation of the Mo surface is enhanced, and an oxide layer with a certain load-bearing capacity is formed, reducing the wear rate of the Mo disk from 135.2 × 10^−5^ to 26.7 × 10^−5^ mm^3^/(N·m). However, local cracking and spalling of the oxide layer keep the friction coefficient relatively high.
(2) In argon, GIS provides good lubrication at RT, with a friction coefficient of 0.27 and a Mo-disk wear rate of 0.90 × 10^−5^ mm^3^/(N·m). As temperature increases, restricted oxidation limits the development of the protective Ga-O-rich tribofilm. Localized Ga retention and enrichment are accompanied by lower near-surface hardness and reduced resistance to plastic deformation, which may promote interfacial weakening, lamellar spalling, and tribofilm instability. At 300°C, the friction coefficient increases to 0.70 and the wear rate rises to 15.6 × 10^−5^ mm^3^/(N·m).
(3) In air, GIS undergoes preferential oxidation and develops a Ga-O-rich composite tribofilm, enabling synergistic lubrication by the liquid-metal layer and the oxidized interfacial film. This composite layer reduces direct Mo/Mo contact and limits direct exposure of the Mo surface to active retained Ga, thereby suppressing Ga-associated interfacial damage. Consequently, GIS lubrication in air exhibits the most stable anti-wear performance over the RT–300°C range. The Mo-disk wear rate remains at 0.8–2.7 × 10^−5^ mm^3^/(N·m), corresponding to a reduction of 90.9%–99.4% compared with dry sliding.

**Figure 13:**
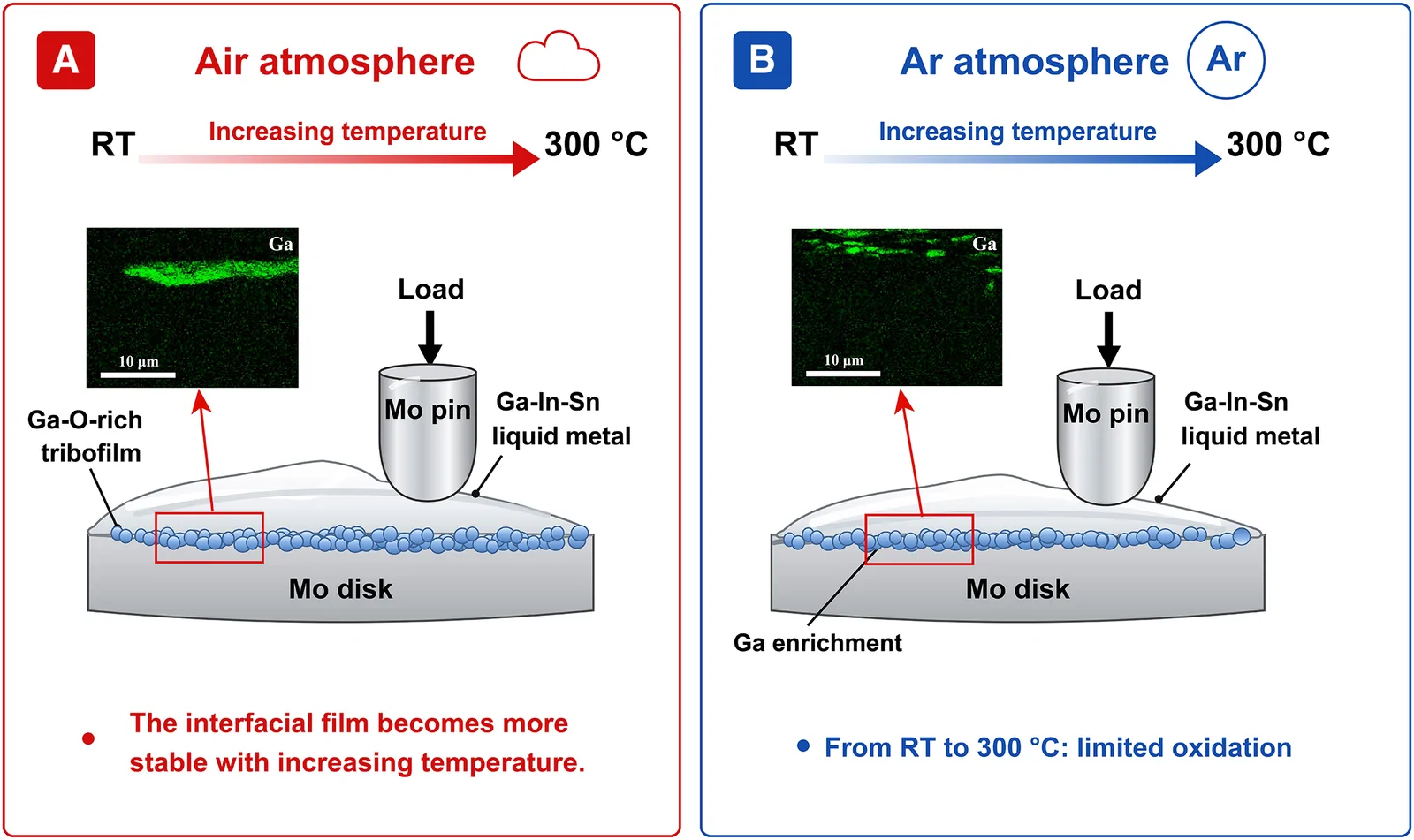
Atmosphere- and temperature-regulated lubrication mechanism of GIS for the Mo/Mo tribopair (A) Air atmosphere. (B) Argon atmosphere. Scale bars: 10 μm in the Ga distribution images.

### Limitations of the study

This study elucidates the atmosphere-regulated lubrication mechanism of a GIS liquid metal lubricant for Mo/Mo tribopairs over a wide temperature range and establishes the relationship between the wear behavior of Mo and the interfacial lubrication behavior of GIS. Nevertheless, several limitations remain. First, the tribological tests were conducted under fixed operating conditions, and the interfacial-film stability under broader loads, sliding speeds, and long-term operation requires further investigation. Second, because pristine-GIS XPS, static oxidation controls, and cross-sectional transmission electron microscopy (TEM)/scanning transmission electron microscopy (STEM) were not obtained, the respective contributions of native, thermal, and friction-assisted oxidation, as well as the nanoscale structure of the tribofilm, cannot yet be independently resolved. Finally, the dynamic evolution of the tribofilm during sliding—including its formation, rupture, and regeneration—as well as the role of Ga in weakening the interface, requires further investigation using *in situ* characterization and mechanical testing.

## Resource availability

### Lead contact

Requests for further information and resources should be directed to and will be fulfilled by the lead contact, Yuan Yu (yuyuan@licp.cas.cn).

### Materials availability

This study did not generate new unique reagents or materials.

### Data and code availability

• All data reported in this paper are available from the lead contact upon reasonable request.
• This study used publicly available commercial software for all data analyses; no custom code was developed or used.
• Any additional information required to reanalyze the data reported in this article is available from the lead contact upon request. If necessary, please contact the corresponding author.

## Acknowledgments

This work was supported by the 10.13039/501100012166National Key Research and Development Program of China (grant no. 2023YFB3405800), the 10.13039/501100001809National Natural Science Foundation of China (52375217), the Taishan Scholars Program of Shandong Province, Shandong Provincial Laboratory Project (SYS202204), Key R&D Program of Shandong Province (Major Innovative and Technological Engineering, grant no. 2024CXGC010316), and Program of Science and Technology Research Project of Higher Education in Hebei Province (CXZX2025021).

## Author contributions

Q.W., conceptualization, methodology, investigation, data curation, formal analysis, visualization, and writing – original draft; H.L., methodology, investigation, data curation, and formal analysis; T.L. and X.F., methodology, investigation, and validation; G.Z., H.C., and Z.M., resources, methodology, and supervision; X.W. and H.T., investigation and validation; Y.Y., conceptualization, methodology, supervision, writing – review and editing, project administration, and funding acquisition; Z.Q., conceptualization, supervision, writing – review and editing, and project administration. All authors read and approved the final manuscript.

## Declaration of interests

The authors declare no competing interests.

## STAR★Methods

### Key resources table

**Table:** 

REAGENT or RESOURCE	SOURCE	IDENTIFIER
**Chemicals, peptides, and recombinant proteins**		

Gallium	Shanghai Maixi Metal Materials Co., Ltd.,China	99.99% Ga
Indium	Shanghai Maixi Metal Materials Co., Ltd.,China	99.99% In
Tin	Shanghai Maixi Metal Materials Co., Ltd.,China	99.99% Sn
Mo disk/Mo pin	Zhuzhou Tongpu Tungsten & Molybdenum New Materials Co., Ltd.,China	Pure Mo

**Other**		

High-temperature friction and wear tester	Lanzhou Zhongke Kaihua Technology Development Co., Ltd., China	HT-1000
Full-color confocal microscope	Leica Microsystems, Germany	DCM8
Energy dispersive spectrometer, scanning electron microscope	TESCAN, Czech Republic	VEGA-GGC001
X-ray photoelectron spectroscopy	Thermo Fisher Scientific, USA	K-Alpha+
X-ray diffractometer	Bruker, Germany	D8 Advance
Nanoindenter	Anton Paar, Switzerland	STeP E400
Vickers hardness tester	Dongguan Kuaijie Measuring Tools & Instruments Co., Ltd., China	HV-30

### Experimental model and study participant details

Omitted as our study does not involve biological models.

### Method details

A ternary Ga-In-Sn gallium-based liquid metal was used as the lubricant in this study, with a mass ratio of Ga:In:Sn = 68.5:21.5:10.0. Representative literature values for near-eutectic Ga_68.5_In_21.5_Sn_10_ (wt. %) include a melting point of approximately 11 °C, a density of approximately 6.44 g/cm^3^, a dynamic viscosity of approximately 2.4 mPa s near RT, and a thermal conductivity of approximately 23–26 W/(m·K).^45^^,^^46^^,^^47^^,^^48^ Ga, In, and Sn were first placed in a conical vessel. The mixture was subsequently heated in an oil bath at 180 °C for 45 min and magnetically stirred under an argon atmosphere to obtain a homogeneous GIS liquid metal.

#### Tribological testing

The tribopair was a Mo disk/Mo pin self-mated pair. The Mo specimens had a nominal purity of 99.95% and an average equivalent grain diameter of 55.12 μm, as determined by EBSD analysis (Figure S12). The specimens were mechanically polished to a surface roughness Ra of 0.02–0.05 μm. Vickers hardness was measured at five locations on the Mo specimens, and the valid measurements were averaged, yielding a mean hardness of 144.86 HV. Tribological tests were conducted on an HT-1000 high-temperature friction and wear tester using a rotating pin-on-disk sliding configuration (Figure S13). The applied load was 10 N, the test duration was 30 min, the sliding speed was 0.13 m/s, and the rotation diameter was 8 mm. Tests were performed at RT, 100°C, 200°C, and 300°C. To investigate the effect of atmosphere on GLM lubrication, three conditions were used: dry sliding in air, GIS lubrication in air, and GIS lubrication in argon.

Before each tribological test, the GIS liquid metal was treated with NaOH solution to remove the pre-existing surface oxide. Fresh GIS was then withdrawn from the interior of the cleaned liquid-metal reservoir using a pipette to minimize reoxidation and contamination during specimen preparation. All experiments used GIS from the same batch and followed identical cleaning and handling procedures. 100 μL of GIS lubricant was introduced into the contact region using a pipette to minimize the influence of lubricant-supply variation. The friction coefficient was recorded in real time by the testing machine. After the test, the three-dimensional morphology and wear volume of the worn region were measured using a Leica DCM8 full-color confocal microscope. The wear rate was calculated as W = V/(F·S), where V is the wear volume, F is the normal load, and S is the total sliding distance. Each test was repeated at least three times, and the average value was used for analysis. After testing, residual liquid metal on the sample surfaces was removed with anhydrous ethanol under water-bath ultrasonic cleaning.

#### Microstructure and composition testing

The worn surfaces of the Mo disks and Mo pins were observed using a VEGA-GGC001 tungsten-filament scanning electron microscope (SEM), and the elemental distribution in the worn regions was analyzed by energy-dispersive spectroscopy (EDS).

The as-prepared GIS was characterized by SEM/EDS and XRD to evaluate its initial elemental distribution and structural state. Cross-sectional SEM and EDS elemental mapping were performed on the worn Mo disks after GIS-lubricated sliding at 300°C in both air and Ar to examine the cross-sectional morphology and elemental distribution. Cross-sectional nanoindentation tests were performed. The maximum indentation load was 15 mN, with loading and unloading rates of 30 mN/min and a holding time of 10 s. Five indentations were performed in both the Ga-affected region and the reference Mo substrate region, and the average mechanical parameters were used for comparison. XPS was conducted on the worn Mo surfaces after GIS lubrication in air at different temperatures and at 300°C in Ar to analyze their surface chemical composition and chemical states.

### Quantification and statistical analysis

The friction coefficient as a function of sliding time was continuously recorded using an HT-1000 tribometer. Figure 2 compares the average friction coefficients of the Mo/Mo tribopair at different temperatures under three conditions: dry sliding in air, GIS lubrication in air, and GIS lubrication in argon. Each average friction coefficient was calculated by averaging all data points collected from the corresponding friction coefficient curve. The wear profiles of the Mo disks were measured using a full-color confocal microscope. The cross-sectional area of each wear track was determined from the measured profile, and the wear volume was calculated by multiplying the average cross-sectional area by the circumference of the wear track. The wear rate was then calculated according to W = V/(F·S), where V is the wear volume, F is the applied normal load, and S is the total sliding distance. Figure 3 presents the wear rates of the Mo disks as a function of temperature under dry sliding in air, GIS lubrication in air, and GIS lubrication in argon.

### Additional resources

This study did not generate additional resources.

During the preparation of this work, the author (s) used ChatGPT for language polishing and sentence revision. The author (s) reviewed and edited the output as needed and take full responsibility for the content of the manuscript.
