# Supplementary material for: Oxidation-regulated wide-temperature tribological behavior and mechanism of a Mo/Mo tribopair lubricated by Ga-based liquid metal
Source: iScience. 2026 Oct 9;29(11):117805. doi: 10.1016/j.isci.2026.117805 (PMC13677202; doi:10.1016/j.isci.2026.117805)
Supplement: Supplementary file 1 [file mmc1.pdf]

iScience, Volume *29*

Supplemental information

Oxidation-regulated wide-temperature tribological behavior and mechanism of a Mo/Mo tribopair lubricated by Ga-based liquid metal

Qiaona Wang, Hongji Liu, Guohua Zhang, Tongyang Li, Hailong Cui, Xing Feng, Yuan Yu, Zhengfeng Ma, Xiaoling Wang, Zhuhui Qiao, and Huaguo Tang

1. Characterization of the as-prepared GIS liquid metal

SEM-EDS analysis shows relatively uniform distributions of Ga, In, and Sn at the micrometer scale, with no obvious elemental segregation or compositionally distinct precipitates (Figure S1).

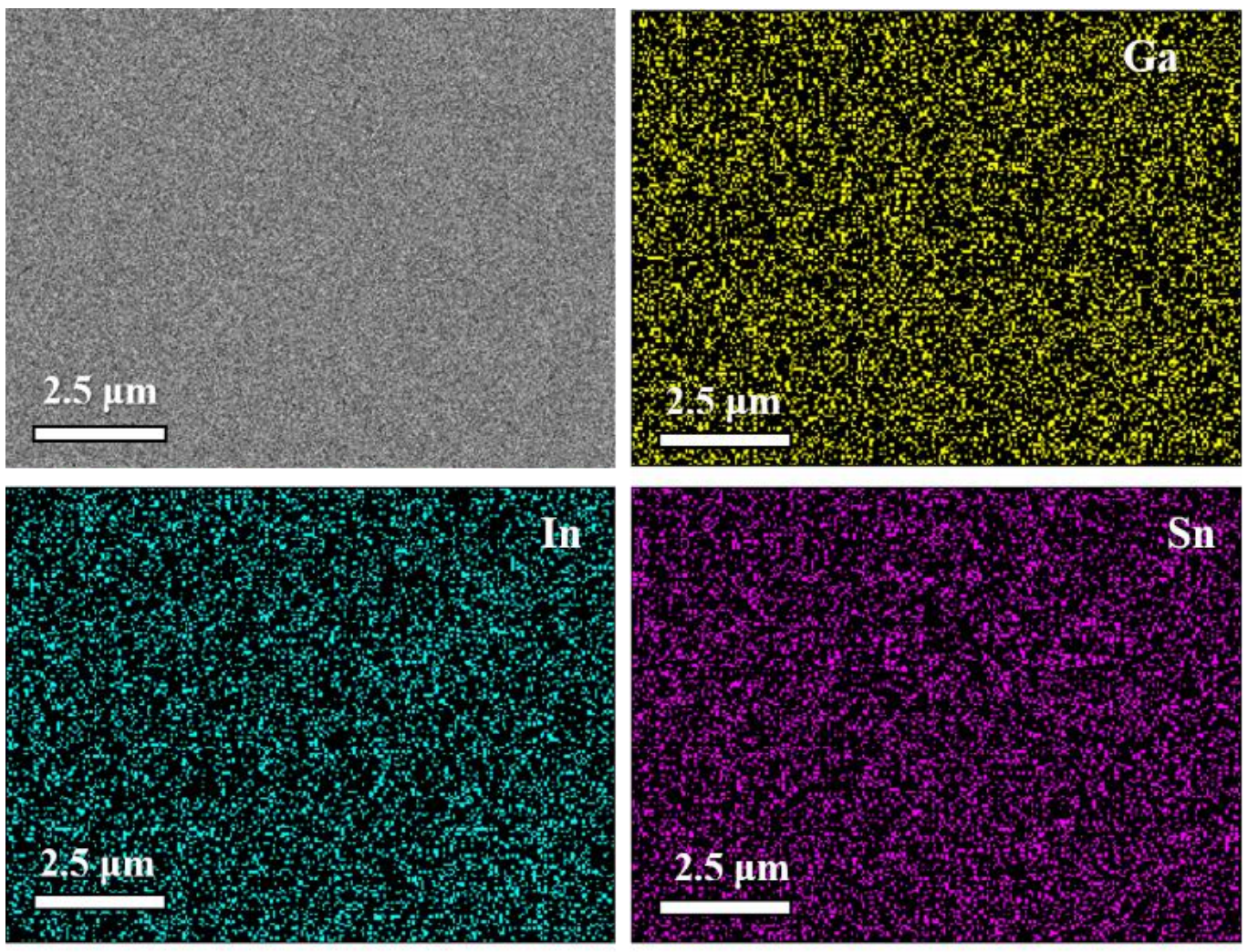

Figure S1. SEM image and EDS elemental maps of the pristine GIS liquid metal. Scale bar: 2.5 μm.

2. Structural state of the as-prepared GIS liquid metal

XRD analysis was performed using a Bruker D8 Advance diffractometer. The pattern exhibits a broad diffuse scattering feature centered at approximately 35° without resolvable sharp Bragg peaks, indicating no detectable long-range crystalline order in the as-prepared GIS liquid metal (Figure S2).

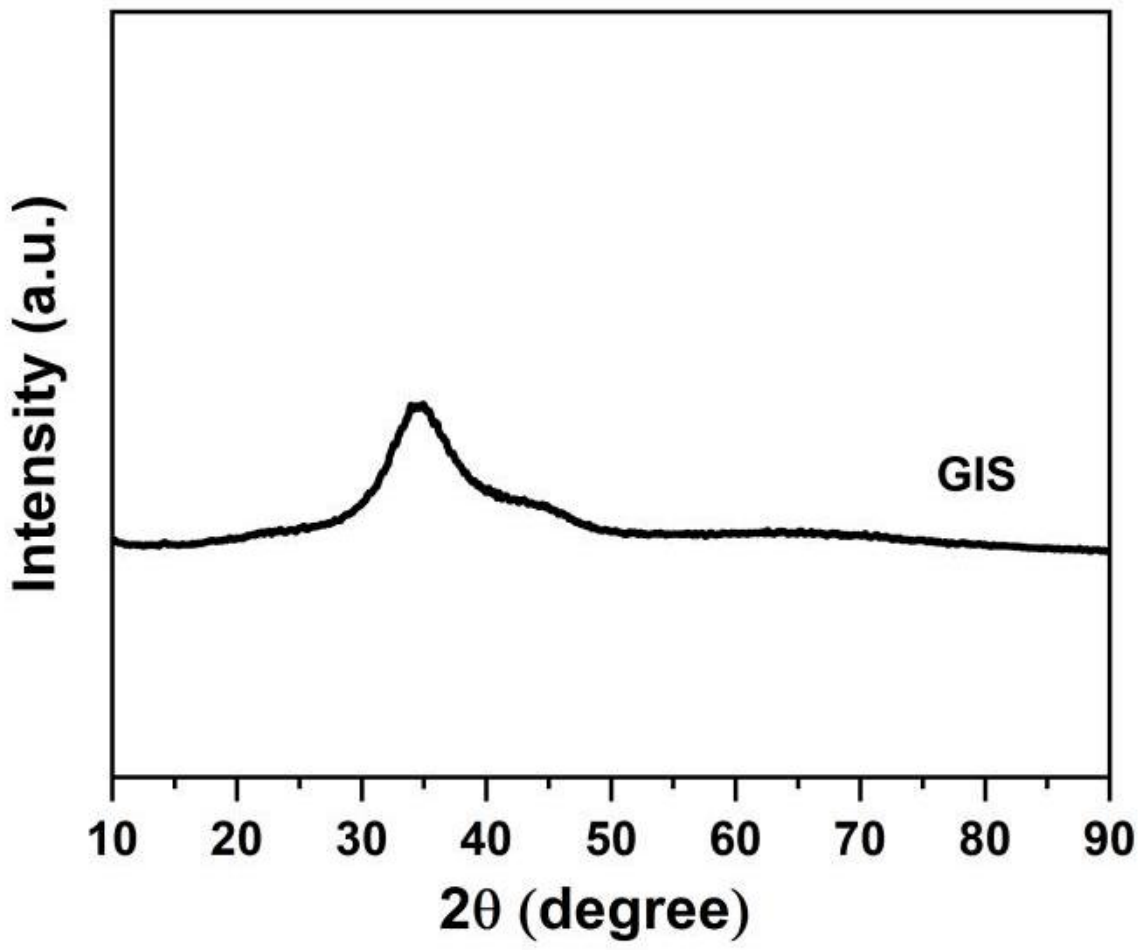

Figure S2. XRD pattern of the pristine GIS liquid metal.

3. Effect of applied load on the tribological behavior of GIS-lubricated Mo

The effect of applied load on the tribological behavior of the GIS-lubricated Mo/Mo tribopairs was further evaluated at 300 ºC in air. As shown in Figure S3, the friction coefficient curves obtained at 7, 10, and 13 N exhibit distinct differences in their frictional behavior. The corresponding average friction coefficients in Figure S4 were 0.815, 0.550, and 0.777, respectively, while the wear rates shown in Figure S5 were 4.8×10^{-5}, 1.7×10^{-5}, and 3.7×10^{-5} $mm^3 \cdot N^{-1} \cdot m^{-1}$, respectively. Compared with 7 N, the friction coefficient and wear rate at 10 N decreased by approximately 33% and 65%, respectively, whereas increasing the load to 13 N resulted in increases of approximately 41% and 118%, respectively.

Overall, the 10 N condition exhibited the lowest friction coefficient and wear rate among the tested loads.

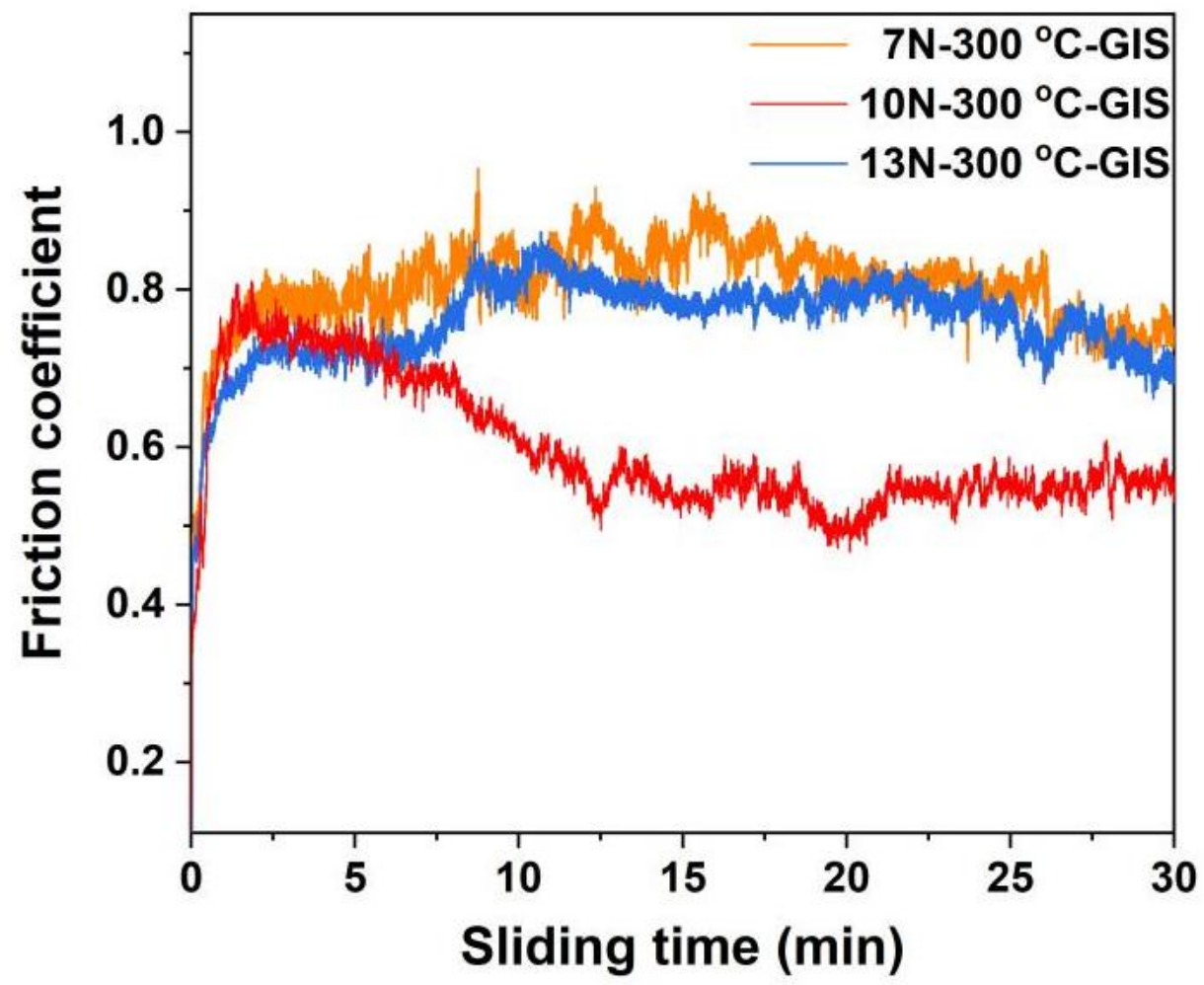

Figure S3. Friction coefficient curves of GIS-lubricated Mo/Mo tribopairs under different loads at 300 °C in air.

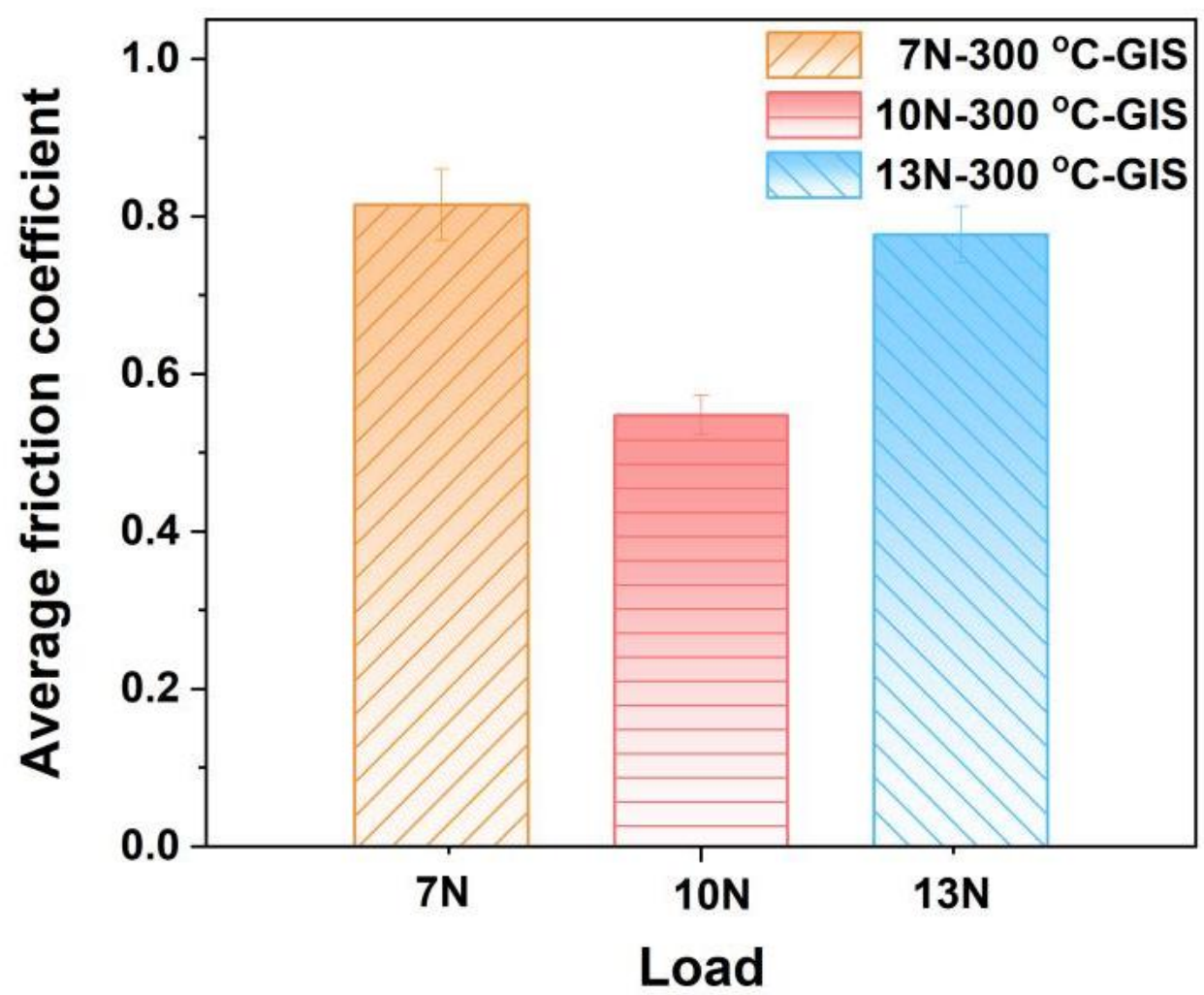

Figure S4. Average friction coefficients of GIS-lubricated Mo/Mo tribopairs under different loads at 300 °C in air. Data are presented as mean values calculated after excluding the initial running-in stage, with error bars representing the variation in the remaining stage.

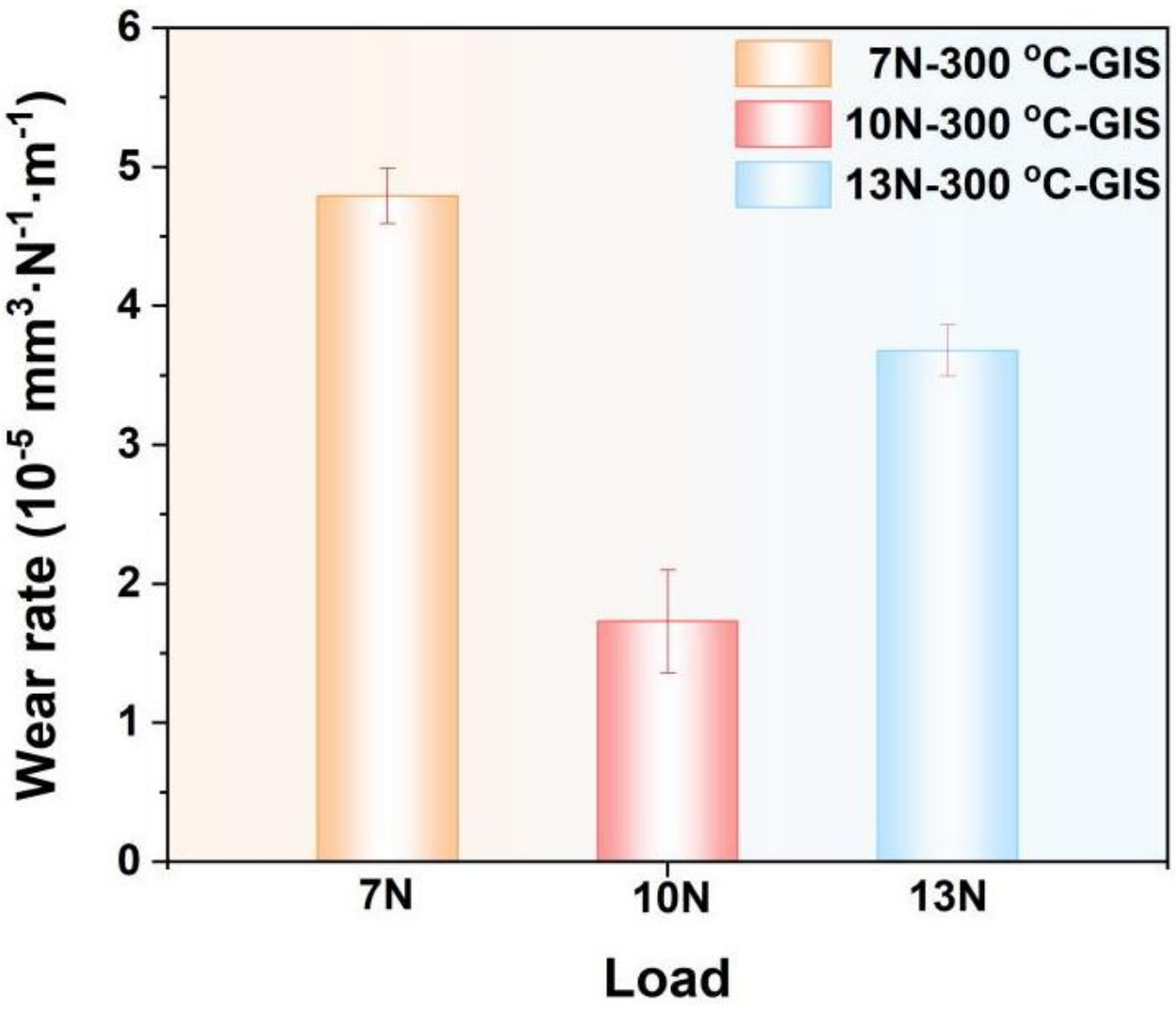

Figure S5. Wear rates of GIS-lubricated Mo/Mo tribopairs under different loads at 300 °C in air. Data are presented as mean ± SD from at least three independent tests.

4. Cross-sectional morphology and elemental distribution of the GIS-lubricated Mo specimen after sliding at 300 °C in Ar

Figure S6 presents the cross-sectional SEM morphology of the worn Mo specimen following GIS-lubricated sliding at 300 °C in Ar. The cross-sectional image shows a relatively shallow deformed and damaged region beneath the worn surface, with localized morphological variations in the near-surface area. Combined with the cross-sectional EDS results shown in Figure S7, Ga is mainly detected near the worn surface and locally extends into the subsurface region of the Mo substrate, indicating localized Ga penetration beneath the sliding surface.

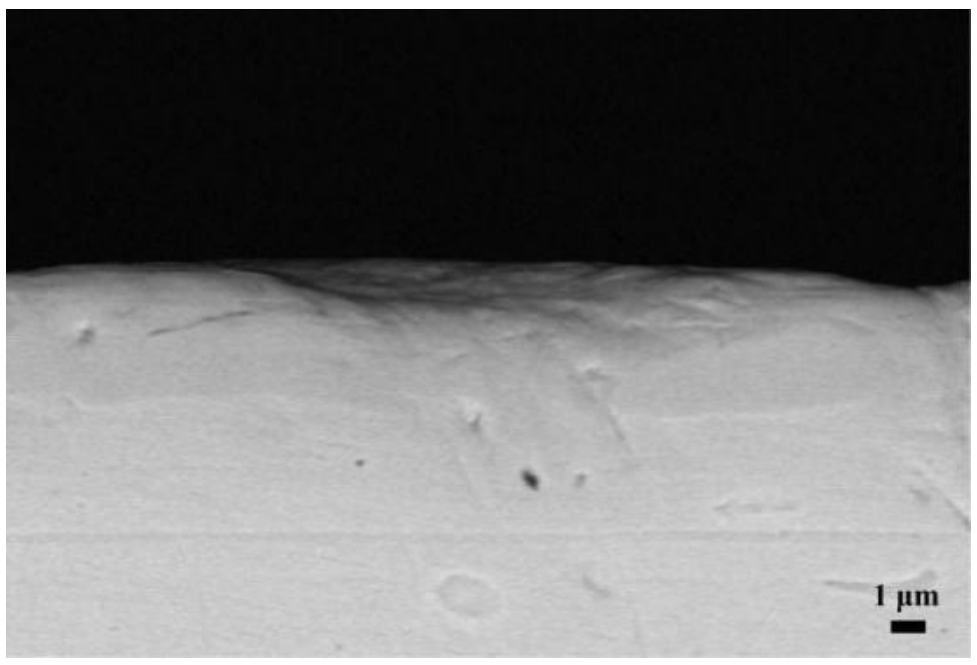

Figure S6. Cross-sectional SEM image of the worn Mo specimen lubricated with GIS at 300 °C in Ar. Scale bar: 1 μm.

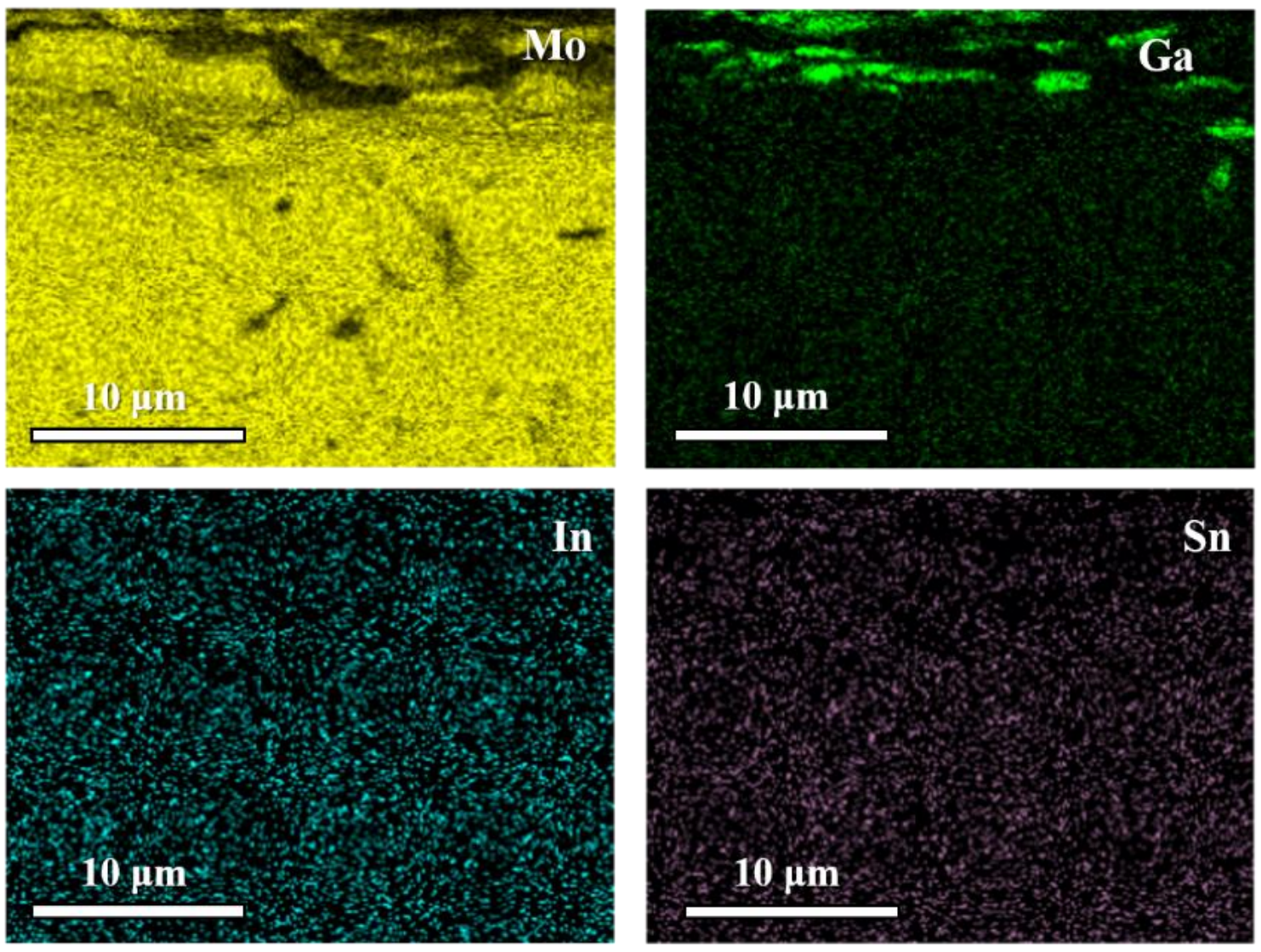

Figure S7. Cross-sectional EDS elemental mapping of the worn Mo specimen lubricated with GIS at 300 °C in Ar. Scale bar: 10 µm.

5. Local mechanical response of the Ga-affected region

Nanoindentation was performed using an Anton Paar STeP E400 nanoindenter. Representative load–displacement responses obtained from repeated measurements show greater maximum and residual penetration depths in the Ga-affected region under nearly identical maximum loads (Figure S8). Table S1 shows lower indentation hardness, H_{IT}/E_r, and H_{IT}^3/E_r^2 values than those of the reference Mo substrate, indicating reduced local hardness, elastic-strain tolerance, and resistance to plastic deformation. These measurements do not directly quantify fracture toughness, ductility, or brittle-fracture susceptibility.

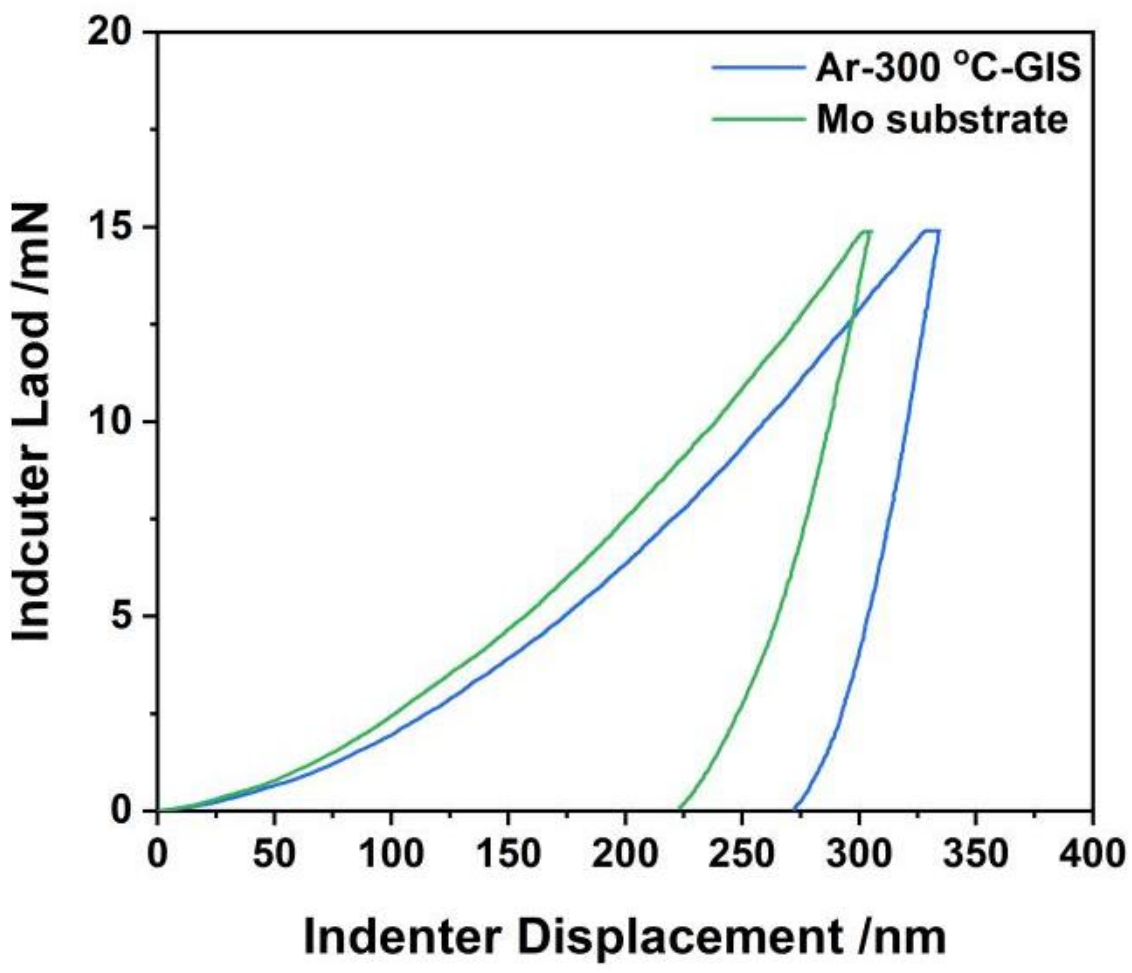

Figure S8. Representative nanoindentation load-displacement curves of the reference Mo substrate region and the Ga-affected region after GIS-lubricated sliding at 300 °C in an Ar atmosphere. Data are presented as mean ± SD from at least three independent tests.

Table S1. Representative nanoindentation-derived mechanical parameters of the reference Mo substrate region and the Ga-affected region after GIS-lubricated sliding at 300 °C in an Ar atmosphere.

Parameter	Symbol	Ga-affected region	Reference Mo substrate region
Indentation hardness	H_{IT}	5.903 GPa	7.625 GPa
Reduced modulus	E_r	208.719 GPa	192.389 GPa
Hardness-to-reduced-modulus ratio	H_{IT}/E_r	0.02828	0.03963
Plastic deformation resistance parameter	H_{IT}^3/E_r^2	0.004722 GPa	0.01198 GPa

6. Cross-sectional morphology and elemental distribution of the GIS-lubricated Mo wear scar after sliding at 300 °C in air

As shown in Figure S9, the cross-sectional SEM image clearly reveals the interfacial morphology between the surface layer of the wear scar and the Mo substrate. Combined with the EDS elemental mapping in Figure S10, the Ga signal is mainly concentrated in the near-surface region and exhibits a relatively continuous distribution along the worn surface, while the Mo signal is predominantly distributed within the underlying substrate. These results indicate that the Ga-containing surface layer is relatively continuously distributed over the examined region.

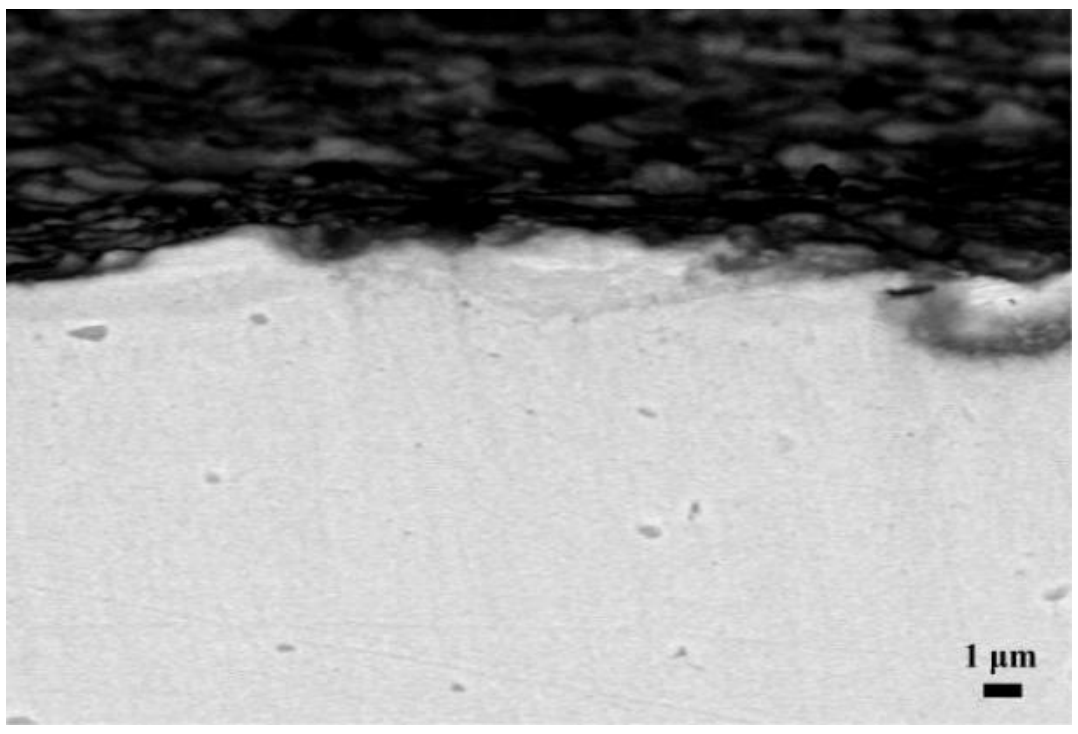

Figure S9. Cross-sectional SEM image of the GIS-lubricated Mo wear scar tested at 300 °C in air. Scale bar: 1 μm.

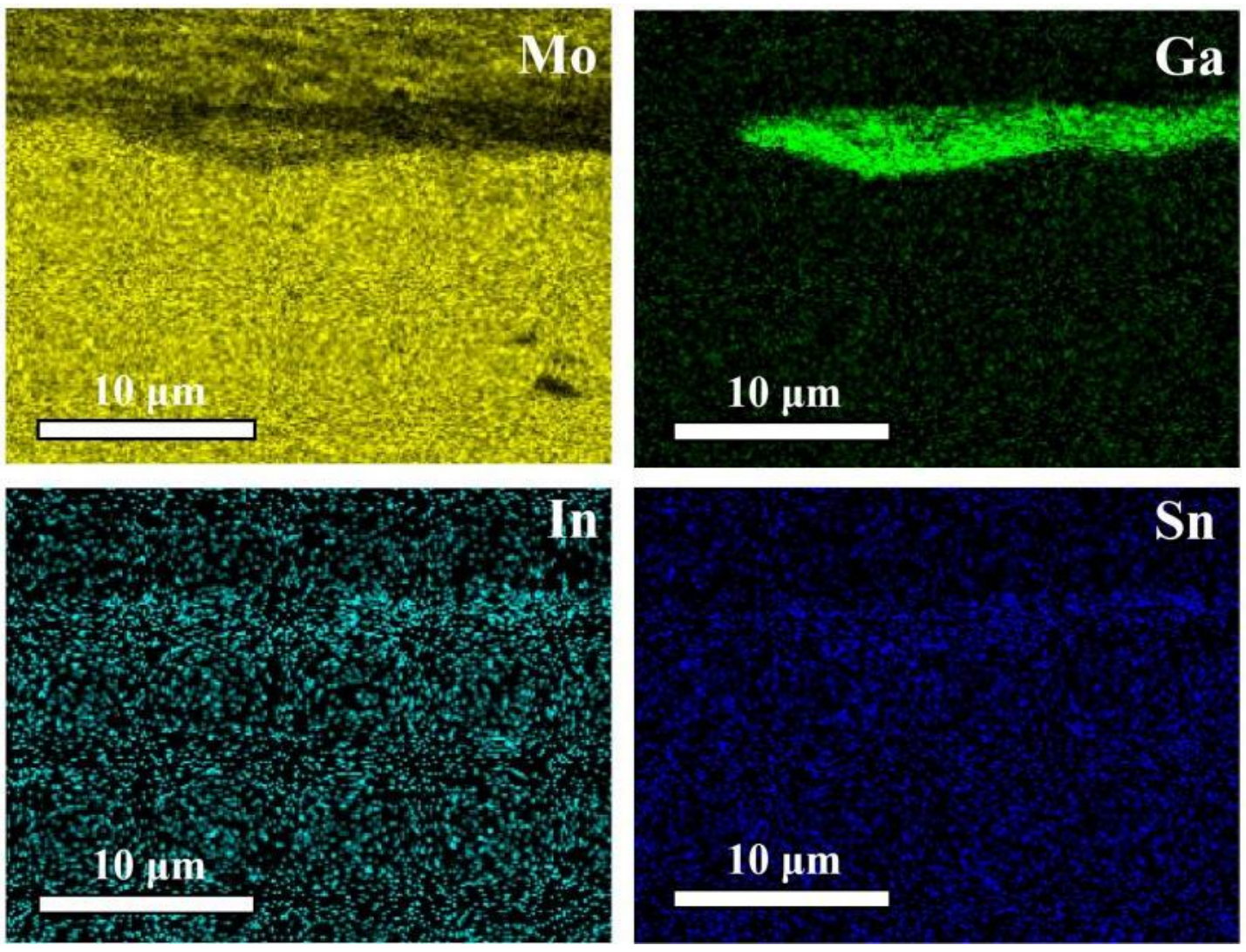

Figure S10. Cross-sectional EDS elemental mapping of the GIS-lubricated Mo wear scar tested at 300 °C in air. Scale bar: 10 μm.

7. XPS characterization of the GIS-lubricated Mo wear scar at 300 °C under Ar

Figure S11 presents the high-resolution XPS spectra of Ga 2p and Mo 3d obtained from the GIS-lubricated Mo wear scar tested at 300 °C in Ar. Both metallic Ga^0 and oxidized Ga^{3+} were detected, with a Ga^{3+}/Ga^0 ratio of 2.86, slightly lower than that under air (3.30), indicating that Ar suppresses but does not completely prevent Ga oxidation. The Mo 3d spectrum retained the characteristic metallic Mo peaks. Combined with the EDS results, these findings indicate that Ga oxidation was partially suppressed under Ar, resulting in a higher Ga content and a lower degree of Ga oxidation on the worn surface compared with air.

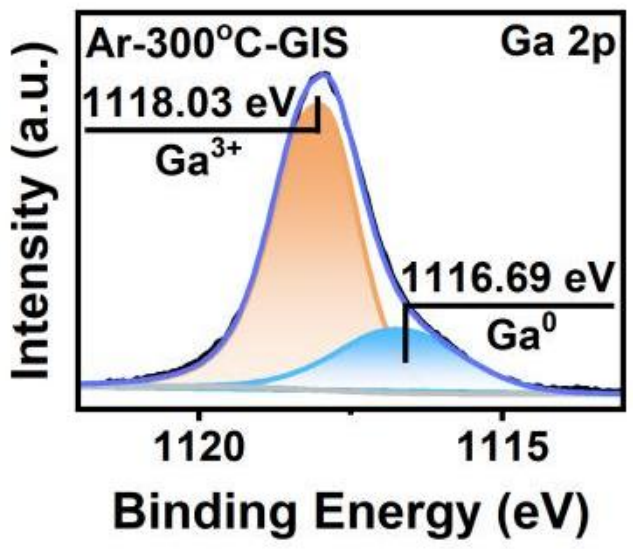

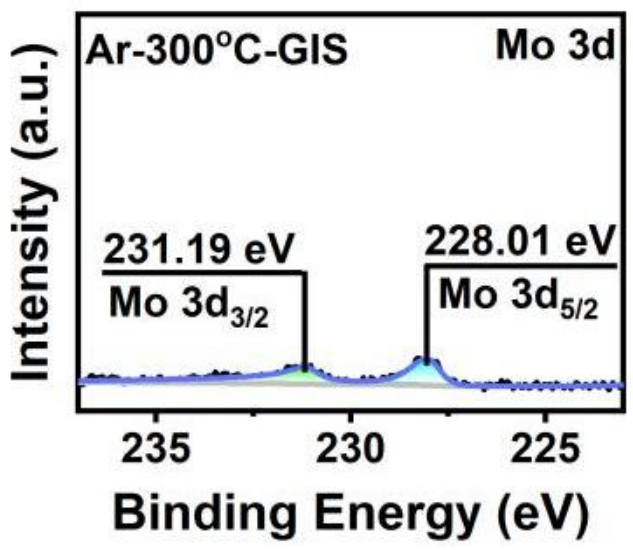

Figure S11. High-resolution XPS spectra of Ga 2p and Mo 3d obtained from the GIS-lubricated Mo wear scars under Ar at 300 °C.

8. EBSD-IPF orientation map of the initial pure Mo specimen

Figure S12 presents the EBSD-IPF orientation map of the initial pure Mo specimen. EBSD analysis yielded an average equivalent grain diameter of 55.12 μm, confirming the grain structure of the specimen in its initial state..

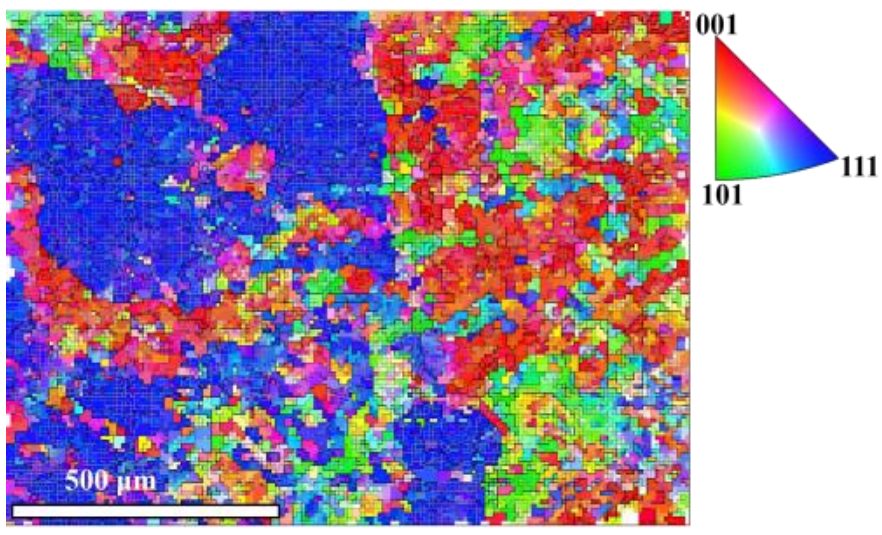

Figure S12. EBSD-IPF orientation map of the initial pure Mo specimen. Scale bar: 500 μm.

9. Schematic illustration of the rotating friction test

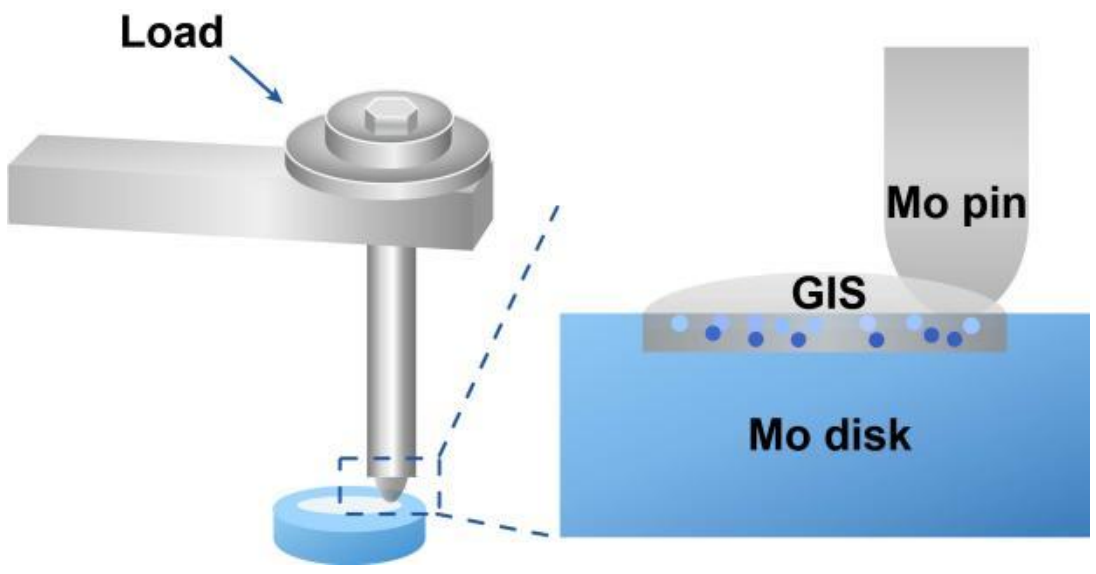

Figure S13. Schematic illustration of the rotating friction test.
